# Isothermal kinase-triggered supramolecular assemblies as drug sensitizers†

**DOI:** 10.1039/c9sc04317a

**Published:** 2019-12-06

**Authors:** Dongdong Liu, Zhe Miao, Chengling Wu, Fangfei He, Peng Ren, Shuo Bai, Xingyu Jiang, Yuan Gao

**Affiliations:** CAS Center for Excellence in Nanoscience, CAS Key Laboratory of Biomedical Effects of Nanomaterials and Nanosafety, National Center for Nanoscience and Technology Beijing 100190 P. R. China gaoy@nanoctr.cn; Sino-Danish College, University of Chinese Academy of Sciences Beijing 100049 P. R. China; Institute of Process Engineering, Chinese Academy of Sciences Beijing 100190 P. R. China; Department of Biomedical Engineering, Southern University of Science & Technology Shenzhen 518055 Guangdong P. R. China

## Abstract

Protein kinases, the main regulators of a vast map of cellular processes, are the most attractive targets in drug discovery. Despite a few successful examples of protein kinase inhibitors, the drug discovery strategy of downregulating protein kinase activity has been quite limited and often fails even in animal models. Here, we utilize protein kinase A (PKA) activity to design PKA-triggered supramolecular assemblies with anticancer activities. Grafting a suitable peptide to PNIPAM raises the critical temperature of the LCST polymer above body temperature. Interestingly, the corresponding phosphorylated polymer has a critical temperature below body temperature, making this peptide-appended PNIPAM a suitable polymer for the PKA-triggered supramolecular assembly process. PKA-triggered assembly occurs selectively in PKA-upregulated MCF-7 cells, which disturbs the cytoskeleton and sensitizes cancer cells against doxorubicin. The chemosensitization is also observed *in vivo* to identify effective tumor inhibitors with satisfactory biocompatibility. Overall, this phosphorylation-induced (in principle, PKA-catalyzed) supramolecular assembly opens up a promising chemotherapy strategy for combating kinase-upregulated cancer.

## Abstract 2

A nonapeptide grafted LCST polymer undergoes enzymatic phosphorylation to assemble, which selectively disrupts PKA overexpressing cancer cells *via* kinetics targeting.

## Graphical abstract

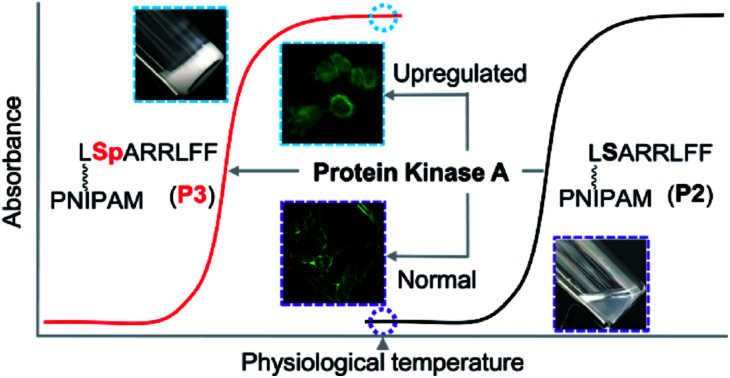

## Introduction

Protein kinases, which constitute almost 2% of human genes, regulate a vast map of cellular processes by mediating the phosphorylation of their substrates.^1^ Because of their central role in signal transduction, protein kinases play causal roles in cancers due to mutations or dysregulation.^2,3^ Therefore, it is a straightforward and attractive strategy to search for protein kinase inhibitors for anticancer purposes,^4–6^ and several drugs have been established.^7^ However, given the strikingly large family of protein kinases, there is a large amount of cross-talk between the enzymes and their substrates (redundant pathways),^8^ making it an arduous task to discover protein kinase inhibitors.^9,10^

Alternatively, enzyme activity could also be used to control cell fate *via* a dynamic process called enzyme-instructed supramolecular self-assembly (EISA). Differentiated by the abnormal activities of certain enzymes in cancer cells compared to normal cells, EISA could selectively occur in or around cancer cells and has shown emerging anticancer activities.^11–14^ By targeting the over-expression of secreted or membrane-bound hydrolases (*e.g.*, alkaline phosphatase, MMPs), the extracellular nanofiber network could block the mass exchange of metabolites in cancer cells.^15^ With additional targeting moieties, such as cholesterol or triphenylphosphine (TPP), cancer-specific EISA could further affect the lipid rafts^16^ or disrupt the normal function of mitochondria.^17^ Moreover, EISA has demonstrated the unique advantage of overcoming drug-resistance issues,^18^ which makes it an emerging anticancer strategy.^14,19^ Nevertheless, thus far, most of the established EISA approaches share a common strategy in which the enzyme converts hydrophilic precursors to hydrophobic assembly building blocks (*e.g.*, phosphatase catalyzed dephosphorylation)^13,20^ and initiates the subsequent self-assembly *in situ*. As the inverse process of dephosphorylation, the phosphorylation that is naturally mediated by a kinase usually induces the disassembly process.^21,22^ Therefore, there has been no example of kinase-triggered self-assembly to control cell fate.

**Scheme 1:**
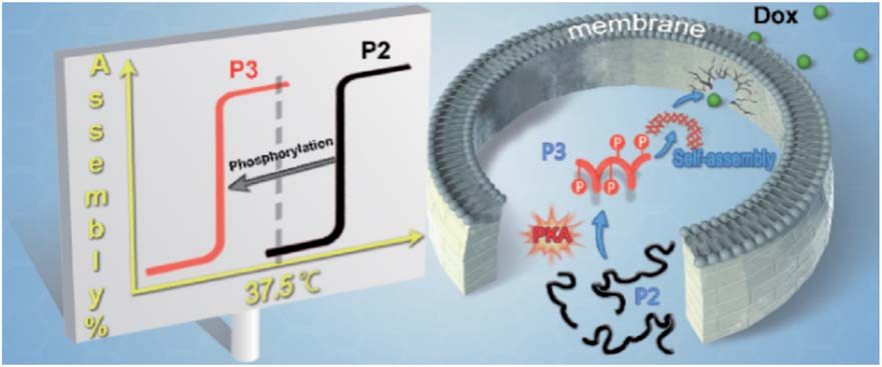
Protein kinase A-triggered intracellular assemblies that can enhance the potency of doxorubicin by weakening the cell membrane.

Since Protein Kinase A (PKA) is closely related to breast cancer,^23^ here, we demonstrate a PKA-triggered isothermal self-assembly process. We designed a nonapeptide derivative (Ac-FFLRRASL-Dpr(COC(CH_3_)=CH_2_)–NH_2_, **P1**) containing a diphenylalanine and a PKA substrate peptide.^24^ A co-polymerization of **P1** and NIPAM yielded a thermoresponsive polymer **P2** with a lower critical solution temperature (LCST). The critical temperature of **P2** was approximately 41.0 °C. Importantly, the corresponding phosphorylated polymer (**P3**) had a lower critical temperature of 36.5 °C, which allowed **P3** to self-assemble and form a hydrogel at 37.0 °C. Therefore, the decrease in critical temperature upon phosphorylation made **P2** a suitable precursor for kinase-induced self-assembly under physiological conditions (*e.g.*, 37.5 °C). Incubation with **P2** induced the selective disturbance of F-actins and microtubules in MCF-7 cells but not MCF-10A cells. Meanwhile, this disturbance of cytoskeletons in MCF-7 cells could be prevented *via* the addition of the kinase inhibitor. The correlation between the disturbances and the over-expression of PKA indicated the formation of PKA-instructed assemblies in MCF-7 cells. Furthermore, we identified the emerging anticancer activity of such assemblies, which acted as a sensitizer of MCF-7 and NCI/ADR-RES cells to doxorubicin, although there was little enhanced toxicity against MCF-10A cells. Overall, we demonstrated a PKA-triggered EISA model that not only broadened the strategy for EISA construction *in situ* but also provided a promising method for anticancer chemotherapy (Scheme 1).

## Results and discussion

**Fig. 1:**
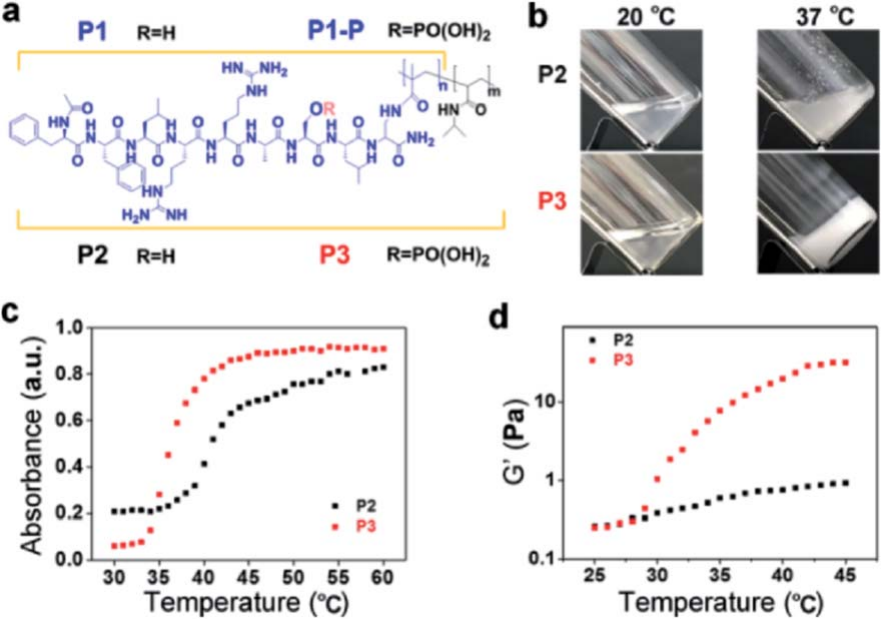
Molecular structures and thermoresponsive behaviour. (a) Molecular structures of **P1**, **P1-P**, **P2** and **P3** (**P2**: *n* = 0.13, *m* = 1; **P3**: *n* = 0.12, *m* = 1). (b) Optical images of the thermoresponsive behavior of **P2** and **P3** (2.0 wt%) between 20 and 37 °C. (c) Temperature-dependent absorbance of **P2** and **P3** (1.0 mg mL^−1^). (d) Temperature-dependent storage modulus values of **P2** and **P3** (1.0 wt%). The temperature was varied from 25 °C to 45 °C with a heating rate of 0.5 °C min^−1^.

Following the typical solid phase peptide synthesis (SPPS) protocol, the peptides **P1** and **P1-P** were synthesized on the Rink Amide AM resin by sequentially conjugating Fmoc–Dpr(ivDde)–OH with suitable Fmoc–amino acids as designed and terminated with the acetyl group (Fig. 1a). After methacryloylation with *N*-methacryloyloxysuccinimide, the peptide derivative **P1**/**P1-P** was purified by reverse-phase high-performance liquid chromatography (RP-HPLC) (Fig. S1 and S2†) and confirmed by electrospray ionization (ESI) MS and ^1^H-NMR. With ammonium persulfate and *N*,*N*,*N*′,*N*′-tetramethylethylenediamine as the redox initiators, the co-polymerization of *N*-isopropyl acrylamide (NIPAM) and **P1** in pure water gave the final product of **P2** with the molecular weight of *M*_n_ = 75 kDa, *M*_w_ = 108 kDa, and PDI = 1.4 as determined by gel permeation chromatography (GPC) (Fig. S3†). The molar fraction of **P1** within **P2** was further identified by ^1^H-NMR. Calculating the integration of the –CH_3_ (*δ* = 1.05 ppm) of *N*-isopropyl acrylamide and –C_6_H_5_ (*δ* = 6–8 ppm) of phenylalanine gave the composition of 11.7 mol% **P1** and 88.3 mol% NIPAM. By replacing **P1** with **P1-P**, **P3** was synthesized with a similar polymerization method. GPC revealed its molecular weight to be *M*_n_ = 77 kDa, *M*_w_ = 136 kDa, and PDI = 1.7 (Fig. S4†). The molar fractions of as-prepared **P3** were also identified by ^1^H-NMR spectroscopy, giving the composition of 11.3 mol% **P1-P** and 88.7 mol% NIPAM.

As a typical LCST thermoresponsive polymer, poly(*N*-isopropyl acrylamide) (PNIPAM) remains soluble in solution when the temperature is below LCST. When the temperature is above LCST, the coil-to-globule transition occurs, and the homogeneous solution becomes a uniformly milky solution.^25^ In our molecular design, we introduced the diphenylalanine (FF) motif, which could promote the supramolecular self-assembly process.^26,27^ Then, we expected supramolecular self-assembly instead of the typical shrinkage of PNIPAM itself.

As shown in Fig. 1b, when heated from 20 °C to 37 °C, the transparent solution of **P2** in PBS (2.0 wt%) turned into a cloudy mixture with a large amount of white precipitate. In contrast, the solution of **P3** in PBS (2.0 wt%) turned into an opaque hydrogel within 3 min when heated to 37 °C. And the critical gelation concentration of **P3** was 6 mg mL^−1^ (Fig. S5†). The UV-vis absorbance also showed the different temperature-dependent behaviour of **P2** and **P3**. As shown in Fig. 1c, the critical temperature of **P2** was 41.0 °C, as determined by the average absorbance at 600 nm between 30 °C and 60 °C.^28^ In contrast, the critical temperature of **P3** was 36.5 °C. Comparing the structural differences between **P2** and **P3** shows that the decrease in the critical temperature should be attributed to phosphorylation. Usually, phosphorylation would improve the water solubility and thus increase the critical temperature.^29^ However, in our case, the net charges of **P1** and **P1-P** are +2 and 0. Therefore, phosphorylation eliminated the electrostatic repulsion, which may have decreased the solubility of the **P1-P**-containing polymer **P3** compared to that of **P2**, resulting in the decrease in the critical temperature.^24^

In addition, the introduction of diphenylalanine strengthened the aromatic–aromatic interactions among the peptides, possibly promoting the intermolecular interaction and supramolecular self-assembly.^30^ In addition to the gelation test in a vial, the temperature-dependent gelation behaviour of **P2** and **P3** (PBS, 1.0 wt%) was monitored using a rheometer (Fig. 1d). In the temperature range of 25 °C to 45 °C (+0.5 °C min^−1^), the storage modulus (*G*′) of **P3** increased significantly at approximately 27.5 °C. In contrast, the *G*′ of **P2** increased quite gently. Rheological frequency sweep tests of **P2** and **P3** were also performed at 25.0 °C, 37.5 °C and 42.5 °C. The frequency dependence of *G*′ and *G*′′ demonstrated the viscoelastic properties of both **P2** and **P3**.^31,32^ However, the *G*′ for **P2** reached a maximum at 37.5 °C and dropped above the critical temperature (*e.g.*, at 42.5 °C), while the *G*′ for **P3** increased monotonically (likely to a plateau above the critical temperature) with increasing temperature (Fig. S6†). These results were well correlated with those shown in Fig. 1b, in which **P2** formed a precipitate but **P3** self-assembled to form a hydrogel.

**Fig. 2:**
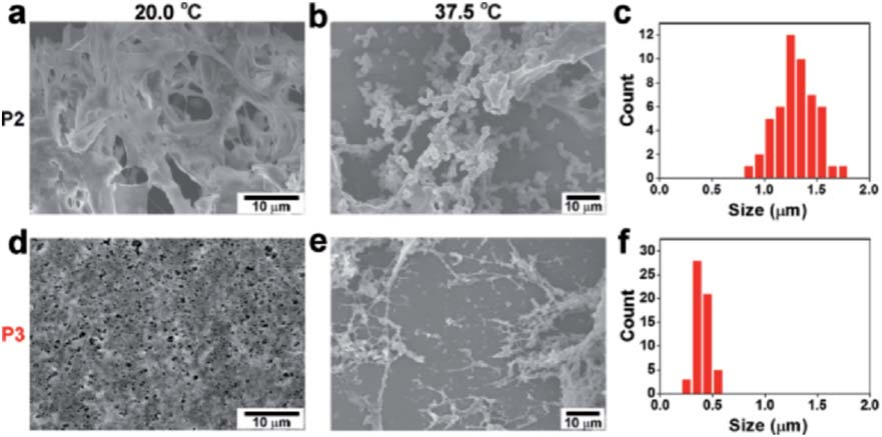
Temperature-dependent self-assembly and nanomorphology. SEM images of **P2** (2.0 wt%) at (a) 20.0 °C and (b) 37.5 °C. (c) Size distribution of the **P2** particles at 37.5 °C. SEM images of **P3** (2.0 wt%) at (d) 20.0 °C and (e) 37.5 °C. (f) Width distribution of the **P3** nanofibers at 37.5 °C.

To elucidate the different assembly phenomena between **P2** and **P3**, the nanomorphology of these temperature-dependent assemblies was inspected by scanning electron microscopy (SEM). At 20.0 °C, both **P2** and **P3** exhibited amorphous structures (Fig. 2a and d). At 37.5 °C, many **P2** microparticles with diameters of approximately 1.3 μm emerged (Fig. 2b–c). When the temperature was increased to 42.5 °C, the **P2** microparticles grew larger, reaching an average diameter of approximately 1.8 μm (Fig. S7a and b†). Since **P3** formed a hydrogel at 37.5 °C, it was not surprising that **P3** formed elongated nanofibres instead of granular particles. The diameter of the **P3** nanofibres was approximately 350 nm (Fig. 2e and f). The nanofibres expanded to approximately 600 nm at 42.5 °C (Fig. S7c and d†). TEM images can also prove that **P2** formed amorphous aggregates, while **P3** and PKA phosphorylated **P2** form fibril nanostructures, which is similar to the morphology observed in SEM images (Fig. S8†). The temperature variance could also affect the CD signals of **P2** and **P3**. Since the diphenylalanine motif often tended to adopt a β-sheet conformation, **P2** gave a negative peak at 213 nm in the soluble state at 37.5 °C. Above the critical temperature, a typical coil-to-globule transition occurred to form microparticles, which resulted in a decrease in the characteristic β-sheet peak at 42.5 °C (Fig. S9b†). In contrast, **P3** exhibited a negative peak at 207 nm once the temperature rose above the critical temperature. The shrinkage of the PNIPAM backbone brought peptides closer, which may have enhanced the β-sheet structure and caused a slight blue shifting, as indicated by the CD signal (Fig. S9c†), resulting in nanofiber formation.^33^ Therefore, at a normal body temperature of approximately 37.5 °C, **P2** and **P3** formed assemblies with drastically different nanomorphologies, which may result in differentiated biological activities (*Vide infra*).

To determine if PKA could phosphorylate our designed molecules, the transfer of phosphate from ATP to serine on **P1** was determined by NMR spectroscopy and mass spectrometry. The PKA catalytic subunit (bought from Merck), ATP and **P1** reacted in Tris–HCl buffer for 4 days, and the mixture was analyzed by analytical HPLC and ESI MS, showing a 76% phosphorylation rate of **P1** (Fig. S10–S14†). The emerging peak in the ^31^P-NMR spectrum could also indicate that PKA can successfully phosphorylate **P1** to yield **P1-P**. Under similar conditions, the PKA catalytic subunit successfully phosphorylated **P2** to yield **P3** according to the HPLC trace and P-NMR spectrum (Fig. S15, S49 and S50†).

To determine whether intracellular PKA could phosphorylate our designed molecules, we cultured MCF-7 cells and MCF-10A cells with **P1** (1.0 mg mL^−1^) for 24 h. Then, we collected the cell lysates to quantify both **P1** and **P1-P** through LC-MS/MS. As shown in Table 1, the amount of internalized **P1** in MCF-7 cells was 3–4 fold more than that in MCF-10A cells, which indicated the higher uptake capability of cancerous cells. The amount of intracellular **P1-P** was almost 8-fold higher in MCF-7 cells than in MCF-10A cells. Overall, the apparent phosphorylation ratios in MCF-7 and MCF-10A cells were 4.6% and 1.9%, respectively. The higher concentration of **P1-P** and phosphorylation ratio could be ascribed to the over-expression of PKA in MCF-7 cells^23^ (Fig. S16†).

**Table 1:** Quantification of **P1** and **P1-P** in MCF-7 and MCF-10A cells pre-incubated with **P1** (1.0 mg mL^−1^) for 24 h

Cell lines	P1/10^4^ cells (nmol)	P1-P/10^4^ cells (nmol)	P1-P/P1 (%)
MCF-7	53.13 ± 0.67	2.42 ± 0.08	4.6%
MCF-10A	16.49 ± 0.28	0.32 ± 0.02	1.9%

**Fig. 3:**
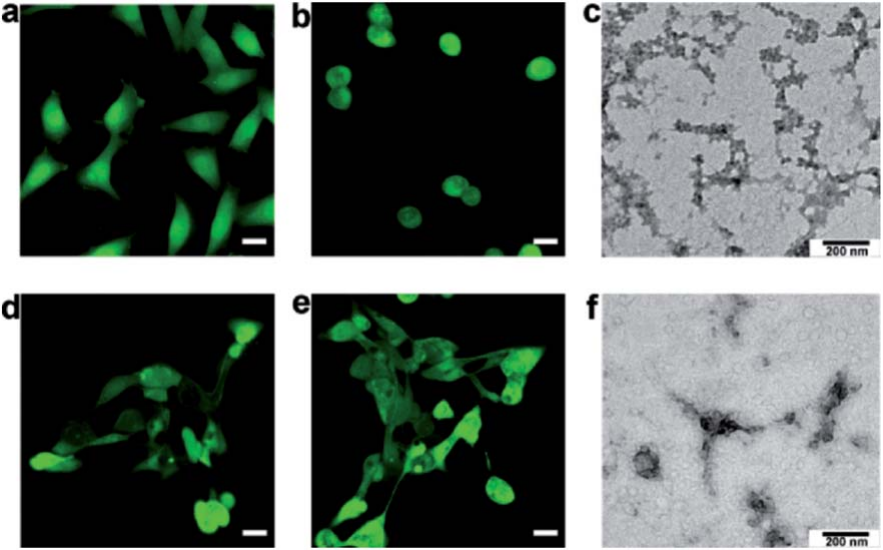
Cell-selective morphological changes due to the formation of intracellular assemblies. MCF-7 cells treated (a) without or (b) with 50 μg mL^−1^**P2** and stained with a cell tracker (2,5-pyrrolidinedione). MCF-10A cells treated (d) without or (e) with 50 μg mL^−1^**P2** and stained with a cell tracker (2,5-pyrrolidinedione). Scale bars: 20 μm. (c) TEM image of the cellular fraction of MCF-7 cells in (b). (f) TEM image of the cellular fraction of MCF-10A cells in (e).

Incubation with **P2** selectively induced morphological changes in MCF-7 cells. As shown by a typical cell tracker, 2,5-pyrrolidinedione, the group of MCF-7 cells pre-incubated with 50 μg mL^−1^**P2** for 24 h exhibited fewer intercellular connections than the wild type group (Fig. 3a and b). We also found that the active PKA was 0.25 U mL^−1^ in MCF-7 cells, 0.04 U mL^−1^ in MCF-10A cells, and 0.005 U mL^−1^ in our solution set-up. The much lower activity *in vitro* thus required a longer time than in living cells to conduct phosphorylation. In contrast, incubation with **P2** did not induce an observable change in cell morphology for cells with normal PKA expression, including MCF-10A cells (Fig. 3d and e) and HUVECs (Fig. S17†). In addition, **P2** did not affect MCF-7 cells with downregulated PKA activity (*e.g.*, by PKA inhibitor H89) (Fig. S18†)^34^ or MCF-7 cells with downregulated expression of the PKA catalytic subunit (Fig. S19 and S20†).^35^ Also, neither **P1′** (Fig. S33 and S41†) nor **P2′** (Fig. S34 and S43†) induced the rounded cellular morphology of MCF-7 cells (Fig. S21†), which further proved that the phosphorylation was a direct mechanism for **P2**-mediated effects in cells. After substitution of acetyl with a fluorophore Cy7 to yield **P2-Cy7**, the confocal images indicated that there was a similar level of fluorescence intensity in both MCF-7 and MCF-10A cells (Fig. S22†). These results indicated that the selective change in the cell morphology of MCF-7 cells was dependent on the upregulated PKA activity. Interestingly, direct treatment with **P3** did not affect the shapes of MCF-7 cells (Fig. S23†). This result implied that **P3** may not efficiently enter MCF-7 cells, as evidenced by the weak fluorescence after incubation with **P3-Cy7** (Fig. S24 and Table S2†). Therefore, **P2** should be efficiently phosphorylated inside MCF-7 cells to yield **P3**, which can then isothermally (at approximately 37.5 °C) assembled intracellularly.

To determine the nanomorphology of intracellular assemblies of **P3** in MCF-7 cells, another group of MCF-7 cells pre-incubated with 50 μg mL^−1^**P2** were lysed in DI water.^36^ As shown in Fig. 3c, there were many self-assembled nanofibers among the MCF-7 cellular fractions. In contrast, there was an absence of self-assembled nanofibers in the cellular fraction of MCF-10A cells pre-incubated with 50 μg mL^−1^**P2** (Fig. 3f). Similarly, no nanofibers were present in the fractions of MCF-7 cells pretreated with **P1** or **P1-P** (50 μg mL^−1^), since neither **P1** nor **P1-P** self-assembled at this concentration (Fig. S25†). Therefore, **P2** was phosphorylated inside MCF-7 cells to yield **P3**, which self-assembled to form nanofibers and induced cell morphological changes thereafter.

**Fig. 4:**
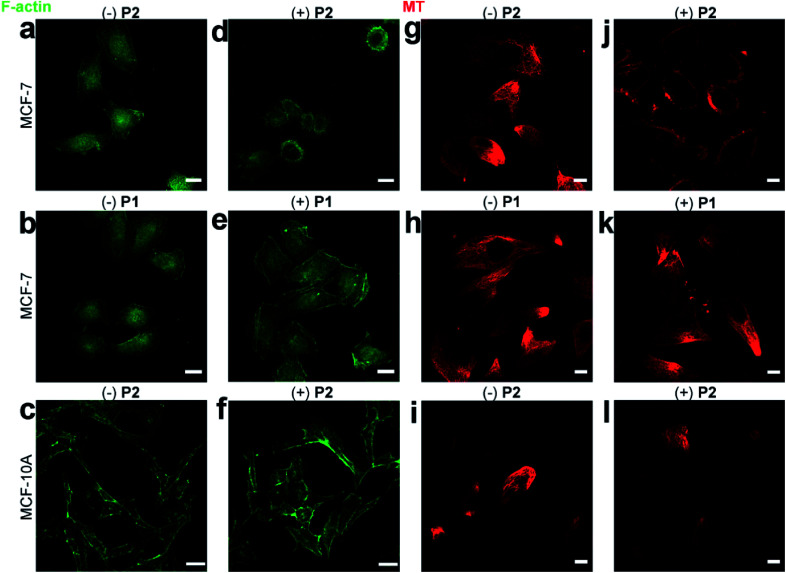
Disturbance of cytoskeletons. FITC-phalloidin-stained F-actins in MCF-7 cells incubated (a) without or (d) with 50 μg mL^−1^**P2**. FITC-phalloidin-stained F-actins in MCF-7 cells incubated (b) without or (e) with 50 μg mL^−1^**P1**. Scale bars: 20 μm. FITC-phalloidin-stained F-actins in MCF-10A cells incubated (c) without or (f) with 50 μg mL^−1^**P2**. Scale bars: 35 μm. Tubulin-Tracker Red-stained microtubules in MCF-7 cells incubated (g) without or (j) with 50 μg mL^−1^**P2**. Tubulin-Tracker Red-stained microtubules in MCF-7 cells incubated (h) without or (k) with 50 μg mL^−1^**P1**. Tubulin-Tracker Red-stained microtubules in MCF-10A cells incubated (i) without or (l) with 50 μg mL^−1^**P2**. Scale bars: 10 μm.

The morphological change in MCF-7 cells was due to the disturbance of cytoskeletons, including F-actins and microtubules.^37^ The disturbance of F-actins was visualized by FITC-phalloidin staining. Consistent with the rounded MCF-7 cells treated with **P2** (Fig. 3b), the F-actin filaments were shortened compared to those of wild-type MCF-7 cells (Fig. 4a and d). Since **P1** did not self-assemble, the F-actins in MCF-7 cells pre-incubated with/without **P1** remained almost identical (Fig. 4b and e). Additionally, the confocal images demonstrated that the F-actins in MCF-10A cells pre-incubated with/without **P2** remained normal (Fig. 4c and f), which was consistent with the normal cell morphology (Fig. 3d and e).

Similarly, the disturbance of microtubules was visualized by Tubulin-Tracker Red staining. Consistent with the rounded cellular morphology of **P2**-treated MCF-7 cells, the microtubules were also shortened compared to those of wild-type MCF-7 cells (Fig. 4g and j). In contrast, **P1** caused little change in the microtubules in MCF-7 cells (Fig. 4h and k). Additionally, the confocal images demonstrated that the microtubules in MCF-10A cells remained normal after incubation with **P2** (Fig. 4i and l).

Although phosphorylation of both **P1** and **P2** required the consumption of ATP, which may affect the homeostasis of the cellular cytoskeleton,^38,39^ only treatment with **P2** induced disturbances in the cytoskeleton. This phenomenon implied that the disturbance in the cytoskeleton was unlikely to be induced by ATP depletion but was dependent on the formation of intracellular assemblies.

Since the cytoskeleton interacts extensively with the membrane,^40^ the disturbance of the cytoskeleton may have further influence on the membrane integrity. The influence of **P2** treatment on the MCF-7 cell membrane integrity was explored with a LIVE/DEAD cell imaging kit.^41^ The results showed that with **P2** treatment, Texas Red not only stains dead cells but also penetrates into the live cells (Fig. S26a†). In contrast, Texas Red did not enter **P1**-treated MCF-7 cells (Fig. S26b†), **P3**-treated MCF-7 cells (Fig. S26c†) or **P2**-treated MCF-10A cells (Fig. S26d†). This observation supported the idea that intracellular self-assembly would increase the MCF-7 cell membrane permeability. The increased membrane permeability of MCF-7 cells will make it easier for **P2** to enter the MCF-7 cells and lead to greater vulnerability under stress.

**Fig. 5:**
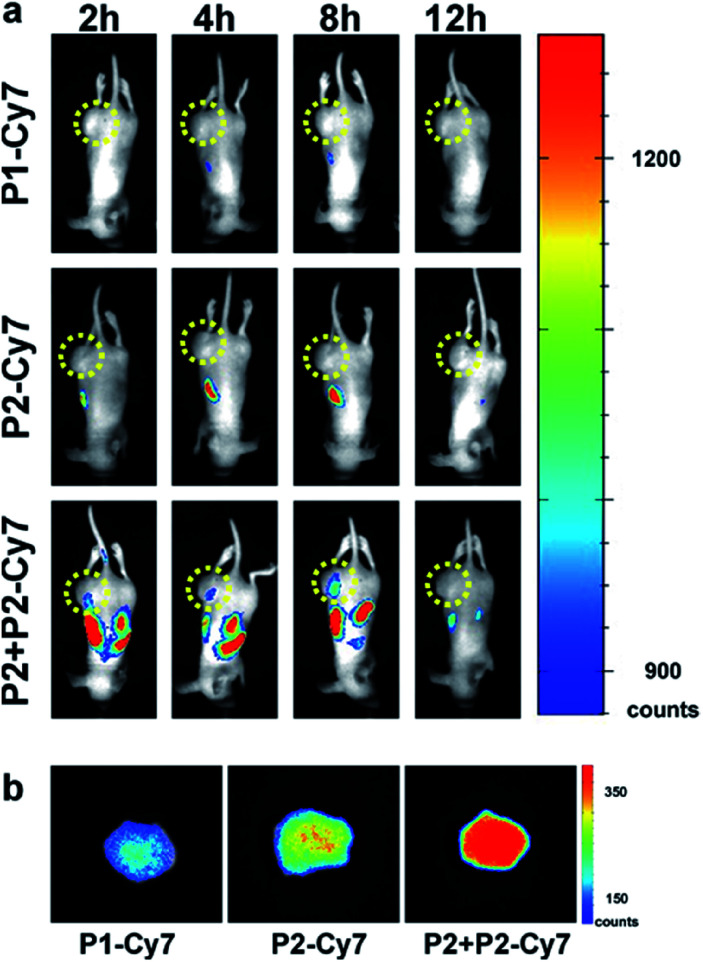
Distribution of **P2**/**P3** in mice. (a) The time-lapse distribution of Cy7 in MCF-7 tumour-bearing mice receiving i.v. injection of 0.50 mg kg^−1^**P1-Cy7**, 0.75 mg kg^−1^**P2-Cy7** or 11.75 mg kg^−1^**P2** + 0.75 mg kg^−1^**P2-Cy7**, respectively. (b) The remaining fluorescence of Cy7 in dissected tumours 24 h post i.v. injection of **P1-Cy7**, **P2-Cy7** or **P2 + P2-Cy7**.

Before the *in vitro* experiments, we have cultured the MCF-7 cells with **P2-Cy7** and **P3-Cy7**. Compared with **P2-Cy7** (Fig. S22a and b†), **P3-Cy7** cannot accumulate into the MCF-7 cells efficiently (Fig. S24†). Besides, the culture of MCF-7 cells with **P3** did not cause an obvious cell morphology change (Fig. S23†). As **P2** showed ideal effects on cells, we paid more attention to the *in vivo* performance of **P2**. To determine if PKA-triggered assembly can target tumours *in vivo*, different groups of MCF-7 tumour-bearing mice received intravenous injections of 0.50 mg kg^−1^**P1-Cy7**, 0.75 mg kg^−1^**P2-Cy7** or 11.75 mg kg^−1^**P2** + 0.75 mg kg^−1^**P2-Cy7**, keeping the same dosage of Cy7 for each mouse according to the fluorescence intensity detected by the *in vivo* imaging system. Then, the spatiotemporal distribution of Cy7 in each mouse was recorded in a time-dependent manner. For **P1-Cy7**, the small molecules were quickly cleared with very weak fluorescence signals detected within the whole mouse. Similarly, little **P2-Cy7** accumulated in the tumour. In contrast, the co-injection of **P2** not only prolonged the half-life of **P2-Cy7** in the mouse but also increased the fluorescence intensity in the tumour (Fig. 5a). At 24 h post-injection, the dissected tumours showed much stronger fluorescence intensity in the **P2 + P2-Cy7** group than in the **P1-Cy7** or **P2-Cy7** group (Fig. 5b). These results demonstrated that **P2** + **P2**-Cy7 was more effectively accumulated and retained longer in tumours than **P1-Cy7** or **P2-Cy7** alone. Since **P1-Cy7** and **P2-Cy7** alone would not assemble at the injected concentration, the retention time of **P2 + P2-Cy7** was prolonged *via* the *in situ* assembly within the tumour.

**Fig. 6:**
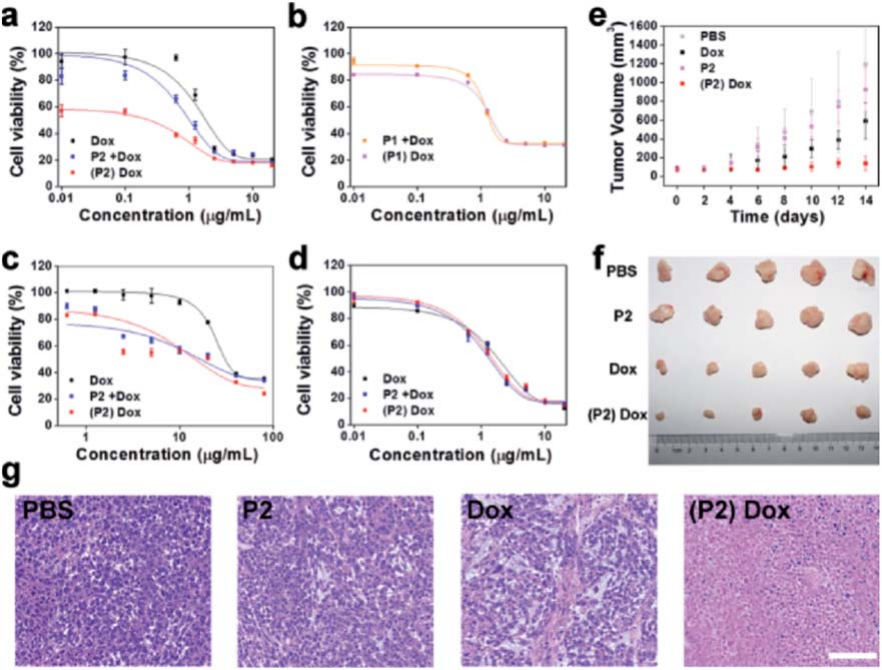
Chemosensitization effect of **P2**. (a) Cell viability of MCF-7 cells in the presence of Dox, **P2** + Dox or (**P2**) Dox. (b) Cell viability of MCF-7 cells in the presence of **P1** + Dox or (**P1**) Dox. (c) Cell viability of NCI/ADR-RES cells in the presence of Dox, **P2** + Dox or (**P2**) Dox. (d) Cell viability of MCF-10A cells in the presence of Dox, **P2** + Dox or (**P2**) Dox. (e) Tumour growth curves of MCF-7 tumour-bearing mice treated with PBS, **P2**, Dox or (**P2**) Dox (*n* = 5). (f) The dissected tumours from the corresponding treatment groups at 14 days. (g) HE staining of the tumours from the corresponding treatment groups. Scale bar: 100 μm.

Although the MTT assay demonstrated that **P2** had no obvious toxicity to MCF-7 and MCF-10A cells (Fig. S27†), the clonogenic assay demonstrated that **P2** selectively decreased the surviving fraction (SF) of MCF-7 cells but not of MCF-10A cells (Fig. S28 and Table S3†). The curtailed division capability of MCF-7 cells may be attributed to the intracellular assembly induced disturbance of the cytoskeleton. This cell selectivity was further tested in a dose–response relationship with doxorubicin. Three treatments were set up as follows: (1) doxorubicin alone (Dox), (2) co-culture of **P2** (100 μg mL^−1^) and Dox (**P2** + Dox), and (3) pretreatment with **P2** (100 μg mL^−1^) for 24 h followed by doxorubicin ((**P2**) Dox). As shown in Fig. 6a, the IC_50_ of Dox against MCF-7 cells was 1.84 μg mL^−1^. In co-culture with **P2**, the IC_50_ decreased to 1.02 μg mL^−1^. Pretreatment with **P2** further decreased the IC_50_ to 0.24 μg mL^−1^. Since **P2** can selectively decrease the surviving fraction of MCF-7 cells, it is not surprising that the (**P2**) Dox group demonstrated a low starting level in the dose–response curve. These results indicate that **P2** can make MCF-7 cells more sensitive to doxorubicin and further enhance the therapeutic effect of Dox.

To determine whether such chemosensitization was due to the assembly process or ATP depletion,^42,43^ the effect of **P1** on the sensitization of MCF-7 cells to doxorubicin was first evaluated. As shown in Fig. 6b, both the co-culture and the pretreatment with **P1** (100 μg mL^−1^) gave IC_50_ values close to that of Dox alone, 1.36 μg mL^−1^ and 1.49 μg mL^−1^, respectively, implying the necessity of assembly. Furthermore, we used the hexokinase-II inhibitor 2-deoxy-d-glucose (2DG) to reduce the intracellular ATP level.^44,45^ Under pretreatment with 2DG (100 μg mL^−1^), the IC_50_ of Dox decreased to 0.99 μg mL^−1^. Conversely, the supplementation of extra ATP (200 μg mL^−1^) to the (**P2**) Dox-treated cells increased the overall IC_50_ from 0.24 μg mL^−1^ to 0.65 μg mL^−1^ (Fig. S29†). According to these ATP manipulation results, **P2** sensitizes MCF-7 cells to doxorubicin *via* both the assembly process and ATP depletion.

The chemosensitization effect of **P2** was valuable for the Dox-resistant cell line NCI/ADR-RES. The IC_50_ of Dox against NCI/ADR-RES cells was 16.73 μg mL^−1^ for the group **P2** + Dox and 14.71 μg mL^−1^ for the group (**P2**) Dox, which were both lower than that of 30.63 μg mL^−1^ for Dox alone (Fig. 6c). In contrast, since **P2** did not form assemblies in MCF-10A cells, the IC_50_ values of Dox were 1.32 μg mL^−1^, 1.47 μg mL^−1^, and 1.83 μg mL^−1^ for the **P2** + Dox, (**P2**) Dox and Dox groups, respectively (Fig. 6d). These similar IC_50_ values indicated that **P2** had no obvious sensitization effect of MCF-10A cells to Dox.

Finally, the chemosensitization effect of **P2** was tested *in vivo*. Four groups of MCF-7 tumour-bearing mice were administered PBS, Dox, **P2**, and (**P2**) Dox separately. Obviously, there was no inhibition effect of PBS or **P2**. However, **P2** could enhance the tumour inhibition capability of Dox. As shown in Fig. 6e, at the dosage of 3 mg kg^−1^, Dox alone cannot inhibit tumour growth within 14 days. The administration of **P2** (10 mg kg^−1^) prior to Dox (3 mg kg^−1^) can effectively inhibit tumour growth eventually (Fig. 6f). Histopathological examination (HE) staining further demonstrated that treatment with (**P2**) Dox caused a higher level of cell apoptosis in tumours than treatment with Dox alone (Fig. 6g). The body weight record (Fig. S30†), the serum biochemical indicator assays (Fig. S31†) and HE staining of the major organs (heart, liver, spleen, lung and kidney) (Fig. S32†) confirmed the satisfactory biocompatibility of **P2**. Overall, the chemosensitization effect of **P2** is promising for the enhancement of the anticancer activity of Dox.

## Conclusion

In conclusion, we demonstrated an example of phosphorylation-induced supramolecular assembly and hydrogelation *via* the combination of self-assembling peptides and the LCST polymer PNIPAM. Usually phosphorylation raised the critical temperature due to the increased hydrophilicity. We utilized phosphorylation to neutralize electrostatic charge to yield a shift towards a lower critical temperature, which was critical to realize the kinase-triggered isothermal supramolecular assembly. Further under open and non-equilibrium conditions such as the biological environment, the assembly process was dependent on the phosphorylation rate which was based on the local and transient concentration of assembling molecules. Such a kinetic selectivity reasoned that the assembly process occurred in PKA-overexpressing MCF-7 cancer cells but not in normal cells. The assemblies disturbed the cytoskeletons and cell membrane and thus enhanced the therapeutic efficacy of doxorubicin by a factor of up to 7.6. Finally, chemosensitization was effective *in vivo* to inhibit tumour growth with satisfactory biocompatibility. Overall, this supramolecular assembly not only provided a new reaction (phosphorylation)-dependent self-assembly mechanism but also selectively impaired PKA-upregulated cancer cells. The capability of supramolecular assembly to remodel cytoskeletons should be useful in suppressing invasion of breast cancer,^46^ increasing intracellular drug concentrations and developing novel anticancer drugs.^47–50^

## Experimental section

### Determination of the LCST of P2 and P3

The LCST of **P2** and **P3** was characterized at a concentration of 1 mg mL^−1^ by 50% of the maximum absorbance at 600 nm. The UV-vis spectra of **P2** and **P3** were obtained using a UV/Vis spectrophotometer (Shimadzu 3600) with a thermoelectric single cell holder (S-1700). A thermoelectric temperature controller was used to control the sample temperature.

### Gelation behaviour of P2 and P3

Rheological measurement was performed on a Kinexus ultra+ rheometer (Malvern Panalytical Technologies) with a standard steel parallel-plate geometry. The PU20 rheometer plate was used in this experiment. **P2** and **P3** were dissolved in PBS (0.5 mL, pH 7.4) at 1 wt% before measurement. The temperature dependent storage moduli (*G*′) of **P2** and **P3** were obtained from 25 °C to 45 °C with a heating rate of 0.5 °C min^−1^ at a frequency of 1 Hz and at a controlled strain of 0.5%.

### Phosphorylation of P1 and P2

The phosphorylation of **P1** was performed according to the protocol given by Merck. In detail, 10 mg **P1**, and 4.57 mg ATP were dissolved in 1 mL buffer (containing 40 mM Tris–HCl and 20 mM magnesium acetate). 10 μg of the PKA catalytic subunit was added to the above solution and maintained in a 30 °C water bath for 4 days. The obtained **P1-P** (76% in yield) was verified by analytical HPLC and ESI MS. Phosphorylation of **P2** was performed under the same conditions as those described above with the same molar ratio of serine.

### Quantification of P1 and P1-P in MCF-10A and MCF-7 cells

To verify that the phosphorylation of **P1** can occur in cells, MCF-10A cells and MCF-7 cells were cultured with **P1** (1.0 mg mL^−1^) for 24 h respectively. And then MCF-7 cells (112 × 10^4^) and MCF-10A cells (188 × 10^4^) were collected and lysed. After lyophilization, the cellular residue was dissolved in 30 mL ultrapure water and methanol (1 : 1). Then the above solution was diluted (1000 times) to the ppb level and the concentrations of **P1** and **P1-P** in MCF-10A cells and MCF-7 cells were tested by LC-MS/MS (Shimadzu, LCMS8050) respectively. The below formula was used to determine the concentration of **P1** and **P1-P** in cells: sample concentration (ppb) × 1000 (dilution ratio) × 30 mL/cell number (10^4^)/molecular weight. The mobile phase for LC-MS/MS consisted of ultrapure water and methanol (1 : 1). The ion pair for determining **P1** was *m*/*z*: 602.95/127.15. The ion pair for determining **P1-P** was *m*/*z*: 642.90/120.05.

### TEM images of the cell fractions

MCF-7 cells were cultured with **P1** (50.0 μg mL^−1^), **P1-P** (50.0 μg mL^−1^), and **P2** (50.0 μg mL^−1^) for 24 h respectively. MCF-10A cells were cultured with **P2** (50.0 μg mL^−1^) for 24 h. Then **P1**, **P1-P** and **P2** containing cell supernatants were removed and the adherent cells were washed with PBS buffer 3 times. After being lysed with 1 mL pure water, the cellular faction was concentrated by centrifugation (300 000*g*, 4 °C, 120 min) and re-dispersed in 200 μL pure water for TEM imaging.

### *In vivo* fluorescence imaging assays of P2

All animal procedures were performed under the supervision and guidance of Animal Care and Use Committee, National Center for Nanoscience and Technology (NCNST). All animal experiments were approved by the Animal Ethics Committee, National Center for Nanoscience and Technology (NCNST) (no. 20184972). The animals were purchased from Beijing Huafukang Bioscience Co., Ltd. For *in vivo* fluorescence imaging assays, female BALB/c nude mice (4 week-old) were injected with 100 μL MCF-7 cells (1 × 10^6^ cells) for constructing the tumour model. 2 weeks after the tumour cells were implanted, tumour-bearing mice were divided randomly into 3 groups. In the treatment group, the mice were injected with 100 μL **P2 + P2-Cy7** (a mixture of 50 μL 4.7 mg mL^−1^**P2** + 50 μL 300 μg mL^−1^**P2-Cy7**) *via* the tail vein. In control groups, the mice were injected with 100 μL 150 μg mL^−1^**P2-Cy7** and 100 μL 100 μg mL^−1^**P1-Cy7***via* the tail vein, respectively. The concentrations of Cy7 in **P2 + P2-Cy7**, **P2-Cy7** and **P1-Cy7** were maintained equal according to the fluorescence intensity detected by the *in vivo* imaging system. The fluorescence images of the mice were monitored after the materials were injected for 2, 4, 8, and 12 h. Then the mice were sacrificed and tumours were taken for fluorescence imaging.

### *In vivo* anti-cancer experiments

The animals were purchased from Beijing Huafukang Bioscience Co., Ltd. To determine the effect of **P2** on improving the chemo-sensitization of Dox, BALB/c nude female mice (around 5 week-old) were subcutaneously implanted with 100 μL (1 × 10^6^) MCF-7 cells. After the tumours grew up to 50–100 mm^3^, tumour-bearing mice were divided randomly into 4 groups (*n* = 5). The mice in control groups were treated with PBS, Dox, and **P2** respectively every three days. The mice in the treatment group were treated with (**P2**) Dox every three days. In detail, PBS and Dox (3 mg kg^−1^) were given *via* the tail vein on the 1^th^, 4^th^, 7^th^, 10^th^, and 13^th^ day. In the group of **P2**, **P2** (10 mg kg^−1^) was directly injected into the tumours on the 1^th^, 4^th^, 7^th^, 10^th^, and 13^th^ day. In the group of (**P2**) Dox, **P2** (10 mg kg^−1^) was directly injected into the tumours on the 0^th^, 3^th^, 6^th^, 9^th^, and 12^th^ day, and Dox (3 mg kg^−1^) was given *via* the tail vein on the 1^th^, 4^th^, 7^th^, 10^th^, and 13^th^ day. During the experiments, the tumour volume and body weight were measured and recorded. A calliper was used to obtain the tumour size and the formula of 0.5 × *ab*^2^ was used to calculate the tumour volume, in which *a* and *b* represent the length and width of the tumour respectively.

## Conflicts of interest

There are no conflicts to declare.

## Supplementary Material

SC-011-C9SC04317A-s001

## References

[cit1] Manning G., Whyte D. B., Martinez R., Hunter T., Sudarsanam S. (2002). Science.

[cit2] Hanahan D., Weinberg R. A. (2011). Cell.

[cit3] Shchemelinin I., Šefc L., Necĉas E. (2006). Folia Biol..

[cit4] Dancey J., Sausville E. A. (2003). Nat. Rev. Drug Discovery.

[cit5] Melnikova I., Golden J. (2004). Nat. Rev. Drug Discovery.

[cit6] Zhang J., Yang P. L., Gray N. S. (2009). Nat. Rev. Cancer.

[cit7] Cohen P., Alessi D. R. (2013). ACS Chem. Biol..

[cit8] Davies S. P., Reddy H., Caivano M., Cohen P. (2000). Biochem. J..

[cit9] Bain J., Plater L., Elliott M., Shpiro N., Hastie C. J., McLauchlan H., Klevernic I., Arthur J. S., Alessi D. R., Cohen P. (2007). Biochem. J..

[cit10] Coleman K. G., Crews C. M. (2018). Annu Rev Cancer Biol..

[cit11] Zhou J., Xu B. (2015). Bioconjugate Chem..

[cit12] Qi G.-B., Gao Y.-J., Wang L., Wang H. (2018). Adv. Mater..

[cit13] Feng Z., Wang H., Chen X., Xu B. (2017). J. Am. Chem. Soc..

[cit14] Yao Q., Lin F., Fan X., Wang Y., Liu Y., Liu Z., Jiang X., Chen P. R., Gao Y. (2018). Nat. Commun..

[cit15] Pires R. A., Abul-Haija Y. M., Costa D. S., Novoa-Carballal R., Reis R. L., Ulijn R. V., Pashkuleva I. (2015). J. Am. Chem. Soc..

[cit16] Wang H., Feng Z., Wu D., Fritzsching K. J., Rigney M., Zhou J., Jiang Y., Schmidt-Rohr K., Xu B. (2016). J. Am. Chem. Soc..

[cit17] He H., Wang J., Wang H., Zhou N., Yang D., Green D. R., Xu B. (2018). J. Am. Chem. Soc..

[cit18] Wang H., Feng Z., Wang Y., Zhou R., Yang Z., Xu B. (2016). J. Am. Chem. Soc..

[cit19] Yao Q., Huang Z., Liu D., Chen J., Gao Y. (2018). Adv. Mater..

[cit20] Kiran S., Hai Z., Ding Z., Wang L., Liu Y., Zhang H., Liang G. (2018). Chem. Commun..

[cit21] Yang Z., Liang G., Wang L., Xu B. (2006). J. Am. Chem. Soc..

[cit22] Zheng Z., Sun H., Hu C., Li G., Liu X., Chen P., Cui Y., Liu J., Wang J., Liang G. (2016). Anal. Chem..

[cit23] Stratakis C. A., Cho-Chung Y. S. (2002). Trends Endocrinol. Metab..

[cit24] Sonoda T., Nogami T., Oishi J., Murata M., Niidome T., Katayama Y. (2005). Bioconjugate Chem..

[cit25] Xia Y., Yin X., Burke N. A. D., Stöver H. D. H. (2005). Macromolecules.

[cit26] Reches M., Gazit E. (2003). Science.

[cit27] Gao Y., Shi J., Yuan D., Xu B. (2012). Nat. Commun..

[cit28] Qiao S.-L., Ma Y., Wang Y., Lin Y.-X., An H.-W., Li L.-L., Wang H. (2017). ACS Nano.

[cit29] Katayama Y., Sonoda T., Maeda M. (2001). Macromolecules.

[cit30] Ma M., Kuang Y., Gao Y., Zhang Y., Gao P., Xu B. (2010). J. Am. Chem. Soc..

[cit31] Wintgens V., Daoud-Mahammed S., Gref R., Bouteiller L., Amiel C. (2008). Biomacromolecules.

[cit32] Antoniuk I., Kaczmarek D., Kardos A., Varga I., Amiel C. (2018). Polymers.

[cit33] Clarke D. E., Pashuck E. T., Bertazzo S., Weaver J. V. M., Stevens M. M. (2017). J. Am. Chem. Soc..

[cit34] Yu M., Liu T., Chen Y., Li Y., Li W. (2018). J. Exp. Clin. Cancer Res..

[cit35] Turnham R. E., Scott J. D. (2016). Gene.

[cit36] Gao Y., Berciu C., Kuang Y., Shi J., Nicastro D., Xu B. (2013). ACS Nano.

[cit37] Fletcher D. A., Mullins R. D. (2010). Nature.

[cit38] Hunter A. W., Caplow M., Coy D. L., Hancock W. O., Diez S., Wordeman L., Howard J. (2003). Mol. Cell.

[cit39] Pollard T. D., Borisy G. G. (2003). Cell.

[cit40] Doherty G. J., McMahon H. T. (2008). Annu. Rev. Biophys..

[cit41] Glisic-Milosavljevic S., Waukau J., Jana S., Jailwala P., Rovensky J., Ghosh S. (2005). Cell Prolif..

[cit42] Kabanov A. V., Batrakova E. V., Alakhov V. Y. (2002). Adv. Drug Delivery Rev..

[cit43] Wang H., Feng Z., Qin Y., Wang J., Xu B. (2018). Angew. Chem., Int. Ed..

[cit44] Kaplan O., Navon G., Lyon R. C., Faustino P. J., Straka E. J., Cohen J. S. (1990). Cancer Res..

[cit45] Kurtoglu M., Gao N., Shang J., Maher J. C., Lehrman M. A., Wangpaichitr M., Savaraj N., Lane A. N., Lampidis T. J. (2007). Mol. Cancer Ther..

[cit46] Padilla-Rodriguez M., Parker S. S., Adams D. G., Westerling T., Puleo J. I., Watson A. W., Hill S. M., Noon M., Gaudin R., Aaron J., Tong D., Roe D. J., Knudsen B., Mouneimne G. (2018). Nat. Commun..

[cit47] Siegal A., Hoenig S., Leibovici J. (1990). Chemotherapy.

[cit48] Huang Y., Sadee W. (2006). Cancer Lett.

[cit49] Stewart M. P., Langer R., Jensen K. F. (2018). Chem. Rev..

[cit50] Hu X., Zhai S., Liu G., Xing D., Liang H., Liu S. (2018). Adv. Mater..

