# Supplementary material for: Isothermal kinase-triggered supramolecular assemblies as drug sensitizers
Source: Chem Sci. 2019 Dec 6;11(4):1132–9. doi: 10.1039/c9sc04317a (PMC8145944; doi:10.1039/c9sc04317a)
Supplement: SC-011-C9SC04317A-s001 [file SC-011-C9SC04317A-s001.pdf]

Electronic Supplementary Material (ESI) for Chemical Science.
This journal is © The Royal Society of Chemistry 2019

Supporting Information

Isothermal Kinase-Triggered Supramolecular Assemblies as Drug Sensitizers

Dongdong Liu, Zhe Miao, Chengling Wu, Fangfei He, Peng Ren, Shuo Bai, Xingyu Jiang and Yuan Gao

Table of Contents

1. Materials and Methods **S2**

2. Chemical Synthesis and procedures **S2**

3. Supplementary Results **S5**

1. Materials and Methods

Rink Amide AM polymer resin, Fmoc-amino acids, and N,N,N',N'-Tetramethyl-O-(1H-benzotriazol-1-yl) uronium hexafluorophosphate (HBTU) were purchased from GL Biochem (Shanghai) Ltd. N^{α}-Fmoc-N^{β}-1-(4,4-dimethyl-2,6-dioxocyclohex-1-ylidene)-3-methyl-buthyl-L-diaminopropionic acid (Fmoc–Dpr (ivDde)-OH) was obtained from Baoman Bio. Acetic acid was purchased from Sinopharm Chemical Reagent Co.,Ltd. Cy7-NHS ester (A8109) was purchased from Apex Bio. FITC-Phalloidin, CellTracker™ Green CMFDA (5-Chloromethylfluorescein Diacetate) - Special Packaging and LIVE/DEAD™ Cell Imaging Kit were purchased from Thermo Fisher. PKA (Catalytic Subunit, Bovine Heart) was purchased from Merck. H89 (dihydrochloride) was purchased from MCE. MTT (3-(4,5-dimethylthiazol-2-yl)-2,5-diphenyltetrazolium bromide) Cell Proliferation and Cytotoxicity Assay Kit was purchased from Beijing Solarbio Science & Technology Co., Ltd. MCF-7, MCF-10A, and NCI/ADR-RES cell lines were purchased from Hunan Fenghui Biotechnology Co., Ltd. Protein kinase A antibody was purchased from Bioss (bs-0520R, Rabbit). GAPDH antibody was purchased from Servicebio (GB12002, Mouse). BCA Protein Assay Kit was purchased from Beyotime. PKA Kinase Activity Assay Kit was purchased from abcom. SignalSilence® PKA C-α siRNA I was purchased from Cell Signaling Technology. PKA α/β cat antibody was purchased from beijing biodragon immunotechnologies co., ltd. EntransterTM-R4000 was purchased from Engreen Biosystem. All the reagents were HPLC grade and used without further purification unless otherwise noticed.

2. Chemical Synthesis and procedures

Synthesis of CH_3COFFLRRASL-Dpr(COC(CH_3)=CH_2)-NH_2 (P1):

We synthesized **P1** through a typical solid phase peptide synthesis (SPPS) with Rink Amide AM resin, corresponding Fmoc-amino acids, Fmoc-Arg(Pbf)-OH, Fmoc-Ser(tBu)-OH, acetic acid and N^{α}-Fmoc-N^{β}-1-(4,4-dimethyl-2,6-dioxocyclohex-1-ylidene) -3-methyl-buthyl-L-diaminopropionic acid (Fmoc-Dpr(ivDde)-OH). For the procedure of SPPS, we used super dried DMF (HPLC grade) as the reaction solvent. We used 20% piperidine in DMF to remove the Fmoc group after each step of coupling. We used HBTU as the coupling reagent. The ivDde group was removed from Dpr with DMF/2% hydrazine for 15 min, and then methacryloylation of the sequence was obtained by coupling it with the N-methacryloyloxysuccinimide in DMF. We used the cleave reagent trifluoroacetic acid (TFA) to cleave the target peptide sequence from the resin (reacted for 6 hours) and then purified it by reverse-phase high-performance liquid chromatography (HPLC) to give **P1** as a white powder (321.0 mg, 26.0%) after lyophilizaiton.

P1 ^{1}H NMR (DMSO-*d6*, 400 MHz) δ (ppm): 8.15-8.04 (m, 3H), 8.04-7.93 (d, 4H), 7.90-7.80 (m, 2H), 7.55-7.45 (m, 2H), 7.27-7.10 (m, 17H), 5.63 (s, 1H), 5.38-5.32 (q, 1H), 4.58-4.48 (d, 1H), 4.47-4.37 (m, 1H), 4.47-4.20 (m, 6H), 4.22-4.15 (t, 1H), 3.63-3.54 (m, 3H), 3.20-2.95 (m, 6H), 2.92-2.75 (m, 2H), 2.70-2.60 (q, 1H), 2.54 (s, 1H), 1.84 (s, 2H), 1.72 (s, 3H), 1.60-1.43 (m, 10H), 1.23-1.15 (t, 8H), 0.90-0.79 (m, 12H). ESI MS (m/z): calcd. for $C_{57}H_{89}N_{17}O_{12}$, 1204.45; found $[M+2H]^{2+}$, 603.1.

Synthesis of CH_3COFFLRRASpL-Dpr(COC(CH_3)=CH_2)-NH_2 (P1-P):

We synthesized **P1-P** through a typical solid phase peptide synthesis (SPPS) with Rink Amide AM resin, corresponding Fmoc-amino acids, Fmoc-Ser(PO(OBzl)OH)-OH, Fmoc-Arg(Pbf)-OH, acetic acid and N^{α}-Fmoc-N^{β}-1- (4,4-dimethyl-2,6-dioxocyclohex-1-ylidene)-3-methyl-buthyl-L-diaminopropionic acid (Fmoc-Dpr(ivDde)-OH). For the procedure of SPPS, we used super dried DMF (HPLC grade) as the reaction solvent. We used 20% piperidine in DMF to remove the Fmoc group after each step of coupling. We used HBTU as the coupling reagent. The ivDde

group was removed from Dpr with DMF/2% hydrazine for 15 min, and then methacryloylation of the sequence was obtained by coupling it with the N-methacryloyloxysuccinimide in DMF. We used the cleave reagent trifluoroacetic acid (TFA) to cleave the target peptide sequence from the resin (reacted for 6 hours) and then purified it by reverse-phase high-performance liquid chromatography (HPLC) to give **P1-P** as a white powder (275.0 mg, 21.0%) after lyophilizaiton.

P1-P ^{1}H NMR (DMSO-*d6*, 400 MHz) δ (ppm): 8.75 (s, 1H), 8.38 (s, 1H), 8.13-8.02 (m, 4H), 7.99 (s, 1H), 7.89-7.82 (m, 2H), 7.62 (s, 1H), 7.34-7.09 (m, 18H), 5.65 (s, 1H), 5.32 (s, 1H), 4.60-4.48 (m, 1H), 4.47-4.40 (m, 1H), 4.40-4.10 (m, 7H), 4.10-3.70 (d, 4H), 3.11-3.02 (m, 6H), 2.92-2.75 (m, 2H), 2.78-2.52 (m, 2H), 2.05-1.95 (q, 1H), 1.83 (s, 3H), 1.71 (s, 3H), 1.65-1.40 (m, 10H), 1.23 (s, 6H), 0.89-0.79 (m, 12H). ESI MS (m/z): calcd. for $C_{57}H_{90}N_{17}O_{15}P$, 1284.42; found $[M-2H]^{2-}$, 641.0.

Synthesis of PNIPAM-PEP (P2):

We synthesized **P2** by a radical copolymerization. Ammonium persulfate and N, N, N', N'-tetramethylethylenediamine were used as the redox initiators. In detail, 40 mg N-isopropylacrylamide (0.35 mmol), 8.1 mg N-methacryloylpeptide (**P1**, 6.7 μmol), 3.1mg ammonium persulfate (2.9 mmol) and 4 μL N, N, N', N'-tetramethylethylenediamine (5.8 mmol) were dissolved in 4.5 mL distilled water and react at 25 °C for 1.5 h under the protection of nitrogen atmosphere. The obtained product was dialyzed for 1-3 days with a semipermeable membrane bag (a molecular weight cutoff of 10 000) against water to give **P2** (29 mg, 60%) after lyophilizaiton.

P2 ^{1}H NMR (DMSO-*d6*, 400 MHz) δ (ppm): 7.50-6.80 (m, -C_6H_5 of **P1**), 1.04 (s, -CH_3 of NIPAM). H(-CH_3):H(–C_6H_5)=4.5.

Synthesis of PNIPAM-PEP-P (P3):

We synthesized **P3** by a radical copolymerization. Ammonium persulfate and N, N, N', N'-tetramethylethylenediamine were used as the redox initiators. In detail, 40 mg N-isopropylacrylamide (0.35 mmol), 8.6 mg N-methacryloylpeptide (**P1-P**, 6.7 μmol), 3.1mg ammonium persulfate (2.9 mmol) and 4 μL N, N, N', N'-tetramethylethylenediamine (5.8 mmol) were dissolved in 4.5 mL distilled water and react at 25 °C for 1.5 h under the protection of nitrogen atmosphere. The obtained product was dialyzed for 1-3 days with a semipermeable membrane bag (a molecular weight cutoff of 10 000) against water to give **P3** (26 mg, 53%) after lyophilizaiton.

P3 ^{1}H NMR (DMSO-*d6*, 400 MHz) δ (ppm): 7.50-6.80 (t, –C_6H_5 of **P1-P**) 1.04 (s, -CH_3 of NIPAM). H(-CH_3):H(–C_6H_5)=4.7.

Synthesize of P1-Cy7

For synthesizing **P1-Cy7**, **P1** (NH_2-FFLRRASL-Dpr(COC(CH_3)=CH_2)-NH_2) was first synthesized by solid phase peptide synthesis (SPPS). **P1** (5.23 mg, 0.0045 mmol), 1.35 μL Diisopropylcarbodiimide (DIPEA) and Cy7-NHS (3.73 mg, 0.0045 mmol) were dissolved in 0.5 mL DMF. The above mixture was stirred at room temperature for 12 h. The product was purified with reverse-phase high-performance liquid chromatography (RP-HPLC) to give **P1-Cy7** as a green powder (2 mg, 22%) after lyophilizaiton.

P1-Cy7 ^{1}H NMR (DMSO-*d6*, 400 MHz) δ (ppm): 8.10-8.05 (t, 4H), 8.04-7.95 (m, 4H), 7.89-7.80 (q, 2H), 7.72-7.65 (q, 3H), 7.59-7.51 (m, 4H), 7.43-7.35 (m, 3H), 7.31-7.11 (m, 15H), 6.18-6.08 (q, 2H), 5.63 (s, 1H), 5.33 (s, 1H), 4.57-4.45 (m, 2H), 4.37-4.21 (m, 5H), 4.22-4.14 (d, 1H), 4.04-4.00 (t, 2H), 3.66-3.57 (d, 19H), 3.11-3.04 (m, 4H), 2.05-1.93 (q, 2H), 1.84-1.79 (t, 4H), 1.70-1.55 (m, 15H), 1.55-1.32 (m, 13H), 1.23-1.17 (m, 10H), 0.88-0.78 (m, 12H).

Synthesize of P2-Cy7

For synthesizing **P2-Cy7**, **P2** (8.94 mg, 0.0045 mmol of amidogen), 1.35 μL DIPEA and Cy7-NHS (3.73 mg, 0.0045 mmol) were dissolved in 0.5 mL DMF. The above mixture was stirred at room temperature for 12 h. Final product can be obtained after purified by dialysis to give **P2-Cy7** (5.5 mg, 43%) after lyophilizaiton.

P2-Cy7 ^{1}H NMR (DMSO-*d6*, 400 MHz) δ (ppm): 7.80-7.00 (m, $–C_6H_5$ of **P1**), 6.25-6.10 (t, -CH of Cy7), 1.05 (s, $-CH_3$ of NIPAM).

Synthesize of P3-Cy7

For synthesizing **P3-Cy7**, **P3** (9.18 mg, 0.0045 mmol of amidogen), 1.35 μL DIPEA and Cy7-NHS (3.73 mg, 0.0045 mmol) were dissolved in 0.5 mL DMF. And then the above mixture was stirred at room temperature for 12 h. Final product can be obtained after purified by dialysis to give **P3-Cy7** (6 mg, 46%) after lyophilizaiton.

P3-Cy7 ^{1}H NMR (DMSO-*d6*, 400 MHz) δ (ppm): 7.60-6.60 (m, $–C_6H_5$ of **P1-P**), 6.25-6.05 (t, -CH of Cy7), 1.05 (s, $-CH_3$ of NIPAM),

MTT assay

MCF-7 cells, NCI/ADR-RES cells and MCF-10A cells were cultured at 37 ^{o}C with a 5% CO_2 humidified incubator. MCF-7 cells were cultured with cell supernatant, containing 90% DMEM, 10% FBS and 1% Pen Strep. MCF-10A cells were cultured with complete medium (purchased from Procell Life Science&Technology Co.,Ltd.). NCI/ADR-RES cells were cultured with cell supernatant, containing 90% DMEM, 10% FBS, 1% Pen Strep, and doxorubicin (1000 ng/mL).

For testing cytotoxicity of **P2**, cells (MCF-7, MCF-10A) were plated into flat-bottom 96-well plates at a density of $0.5\text{-}1\times10^4$ cells per well and cultured for 12 h. Then, cell culture medium was replaced with cell culture medium containing **P2** and cultured for 72 h. Cell proliferation was analyzed with a standard MTT assay.

For testing chemo-sensitivity effect of **P2**, cells (MCF-7, MCF-10A, NCI/ADR-RES) were plated into flat-bottom 96-well plates at a density of $0.5\text{-}1\times10^4$ cells per well and cultured for 12 h. Then, cell culture medium was replaced with Dox (doxorubicin alone), or **P2**+Dox (co-culture of 100 μg/mL **P2** and Dox), or (**P2**) Dox (pre-treatment with 100 μg/mL **P2** for 24 hours and then Dox) respectively. Cell proliferation of each group was analyzed with a standard MTT assay after 24 h's incubation.

Clonogenic assay

Following a standard clonogenic assay protocol, MCF-7 cells were treated with **P2** (0, 25, 50, 100 μg/mL) for 24 h and then we collected the pre-treated cells, suspended them in fresh culture medium. After seeding into 6 well plates at the density of 200, 400, 800 cells/well, we further cultured these cells for 14 days at 37 ^{o}C with a 5% CO_2 humidified incubator. MCF-10A cells were treated with **P2** (0, 25, 50, 100 μg/mL) for 24 h and then we collected the pre-treated cells, suspended them in fresh culture medium. After seeding into 6 well plates at the density of 1600, 3200, 4800 cells/well, we further cultured these cells for 14 days at 37 ^{o}C with a 5% CO_2 humidified incubator. 14 days later, the culture medium was removed carefully and washed with PBS for 3 times. Then the cell colonies were fixed with 6.0% glutaraldehyde (vol/vol) and stained with 0.5% crystal violet (wt/vol). The colonies with >50 cells were counted. Plating efficiency (PE) and surviving fraction(SF) were calculated with the formula given below.

PE=number of colonies formed/number of cells seeded × 100%.

SF=number of colonies formed after treatment/(number of cells seeded × PE).

Western blot experiment

The cultured MCF-7 cells and MCF-10A cells were washed with PBS and collected with cell scraping. The

collected cells were lysed with Ripa lysate (Servicebio, G2002) on ice for 30 minutes. For collecting the supernatant, the cell lysate were centrifuged at 12000 rpm, 4 °C for 10 min. Protein concentration was determined by the BCA Protein Assay Kit. 5% SDS-PAGE was prepared and used to separate the proteins, and then the separated proteins were transferred to pre-activated PVDF membranes. 5% nonfat milk (Servicebio, G5002) was prepared freshly and was used to block the blots for 1 hour. After the membrane were incubated with PKA antibody at 4 °C overnight, we used TBST to wash it for 3 times, the membrane were then incubated with HRP labeled secondary antibody at room temperature for 0.5 h. With another washing with TBST for 3 times, the immunoblots were analyzed in dark room.

Determination of the active PKA concentration

4×10^6 MCF-10A cells and 7.5×10^5 MCF-7 cells were collected and resuspended in 500 µL lysis buffer for 30 minutes on ice respectively. The clear supernatant was collected respectively after centrifuge lysate at 13,000 rpm for 15 minutes. Then the active PKA was determined according to the protocol given by PKA Kinase Activity Assay Kit. The active PKA was determined to be 503.55 ng per 10^4 MCF-7 cells, 78.97 ng per 10^4 MCF-10A cells. The active PKA in the PKA catalytic subunit (bought from Merck) is 148.38 ng per µg PKA catalytic subunit. The active PKA offered by the PKA Kinase Activity Assay Kit was 0.50 U/mg and the volume of the cells were around 100 fL per cell. So the results demonstrated that the active PKA concentration was 0.25 U/mL in MCF-7 cells, 0.04 U/mL in MCF-10A cells, and 0.005 U/mL in our solution set-up, respectively.

3. Supplementary Results

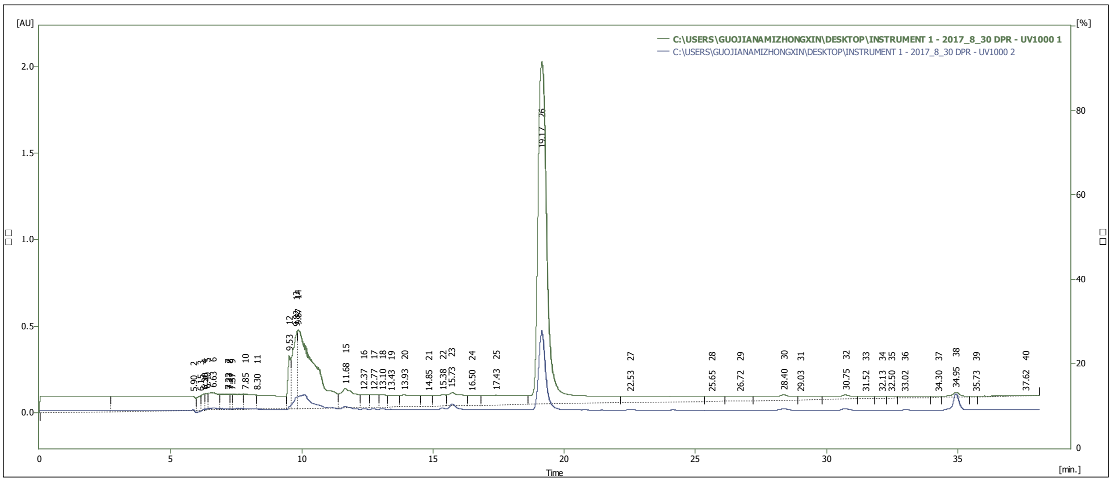

Figure S1. HPLC spectrum of **P1**.

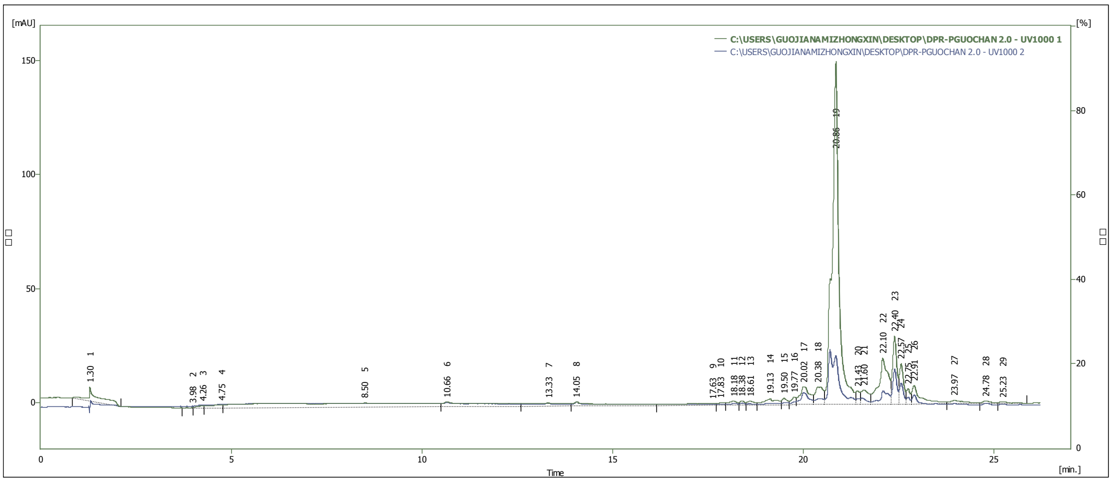

Figure S2. HPLC spectrum of **P1-P**.

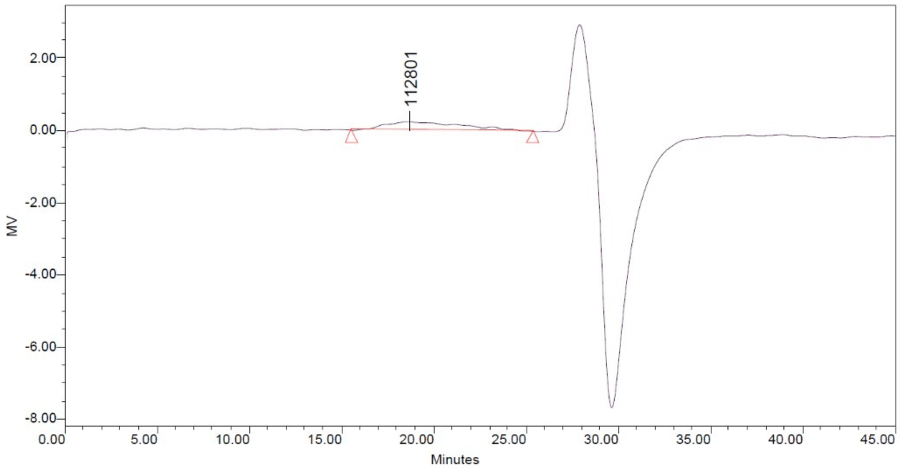

Figure S3. Autoscaled chromatogram of **P2** obtained by gel permeation chromatography.

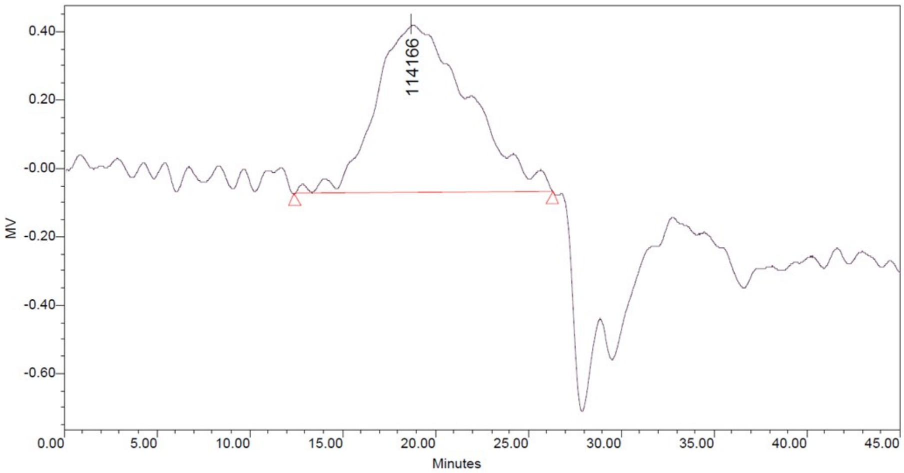

Figure S4. Autoscaled chromatogram of **P3** obtained by gel permeation chromatography.

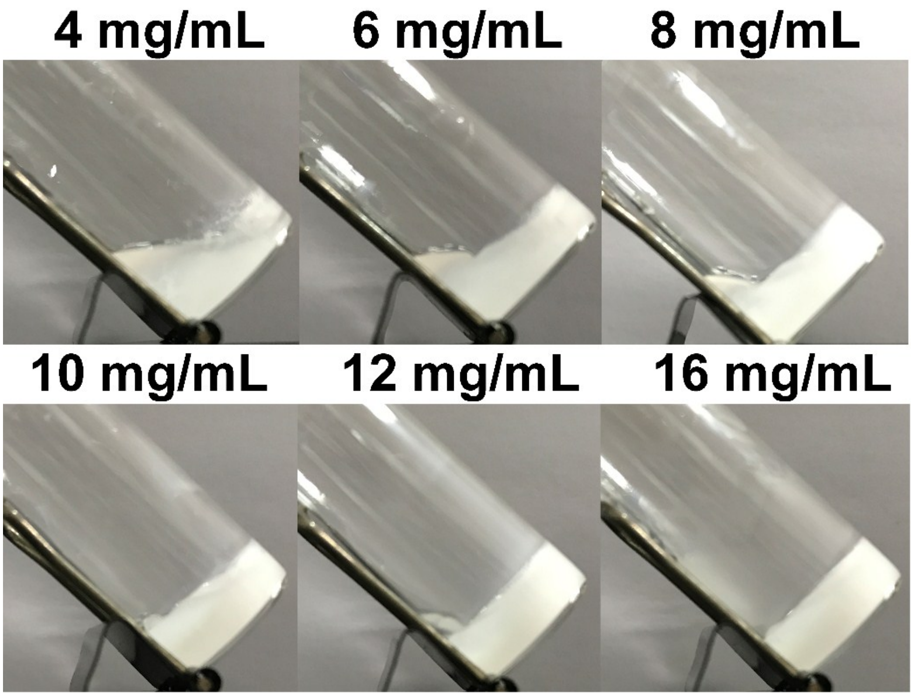

Figure S5. Determination of the critical gelation concentration of **P3** in PBS at 37.5 °C.

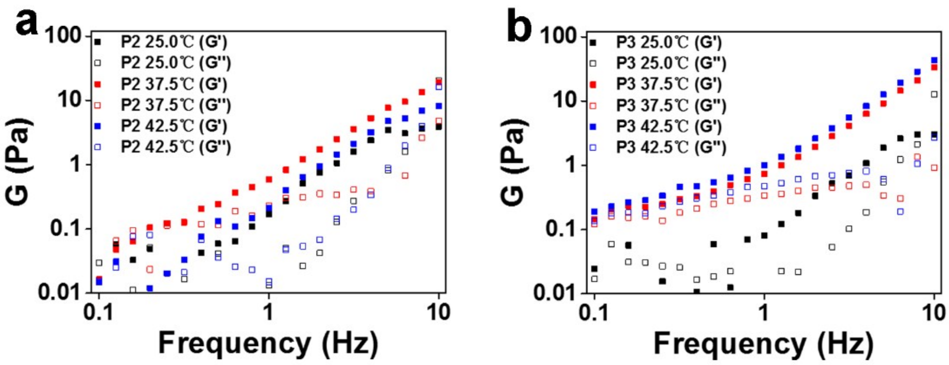

Figure S6. Storage modulus (G′) and Loss modulus (G ") of **P2** (a) and **P3** (b) (1.0 wt%) at 25.0 °C, 37.5 °C and 42.5 °C at a controlled strain of 0.5%.

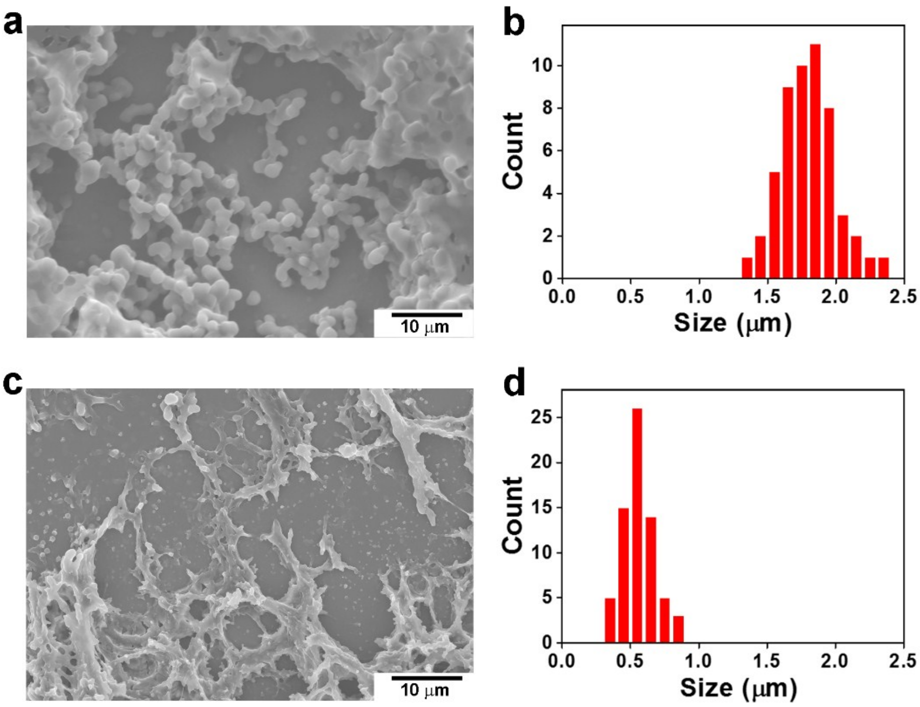

Figure S7. SEM image of **P2** at 42.5 ^{o}C (a) and the size distribution of the **P2** particles (b). SEM image of **P3** at 42.5 ^{o}C (c) and the width distribution of the **P3** fibers (d) Scale bar: 10 μm.

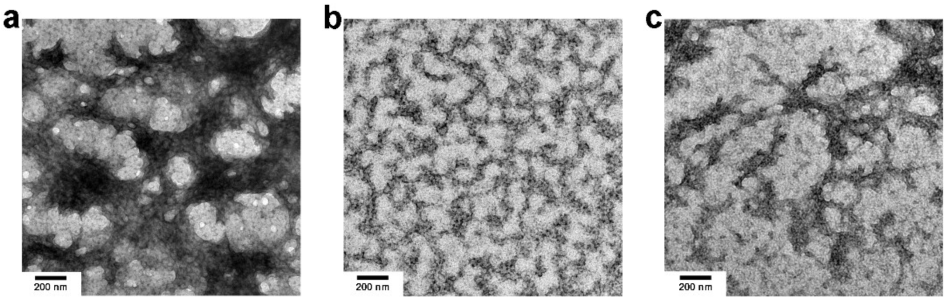

Figure S8. TEM images of (a) **P2**, (b) **P3** and (c) **P2** treated with PKA for 4 days at 37.5 ^{o}C. Scale bars: 200 nm.

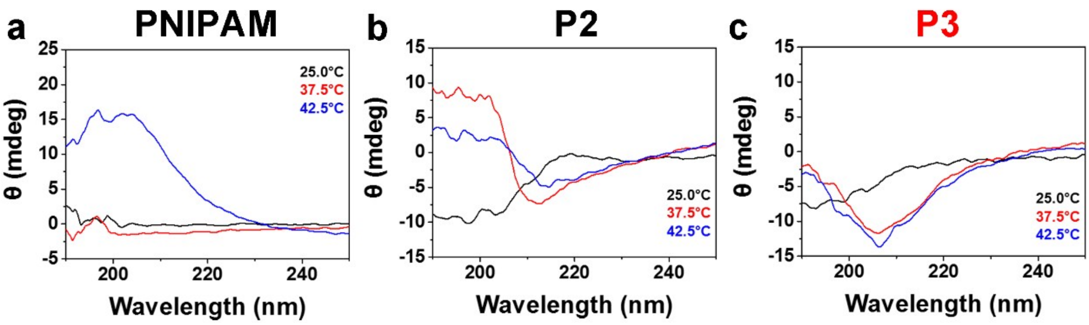

Figure S9. CD spectra of (a) PNIPAM, (b) **P2** and (c) **P3** (50 μg/mL) at 25.0 °C, 37.5 °C and 42.5 °C.

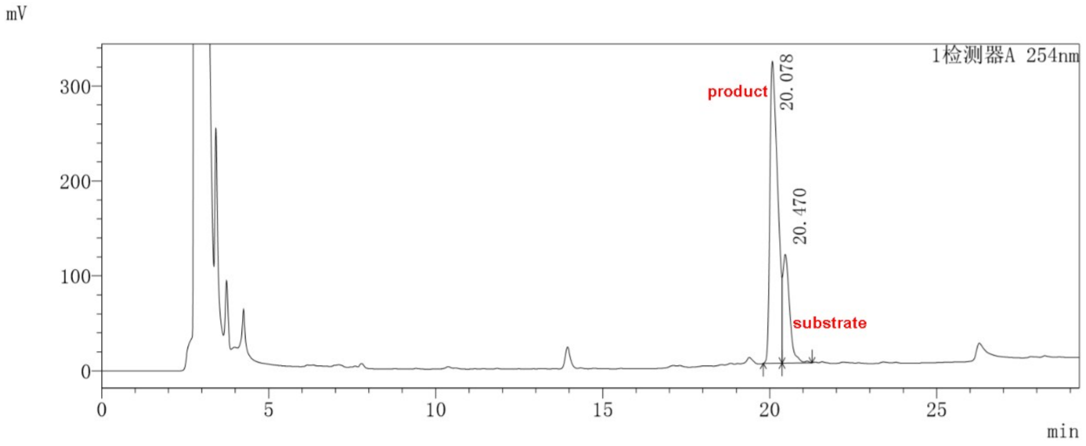

Figure S10. HPLC trace of **P1** after phosphorylated by PKA catalytic subunit for 4 days, **P1** is the substrate, **P1-P** is the product.

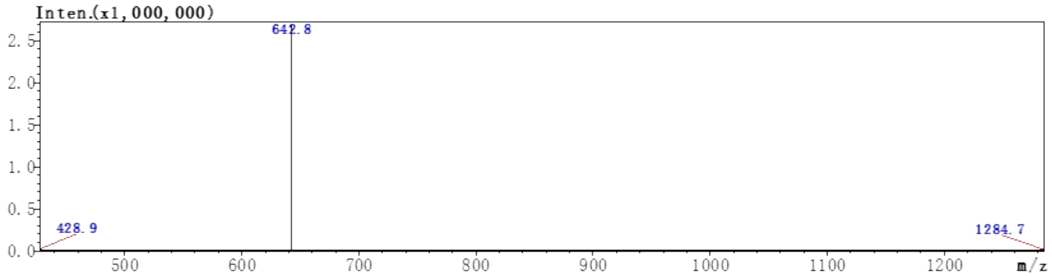

Figure S11. ESI MS spectrum of product peak (**P1-P**) in Figure S10.

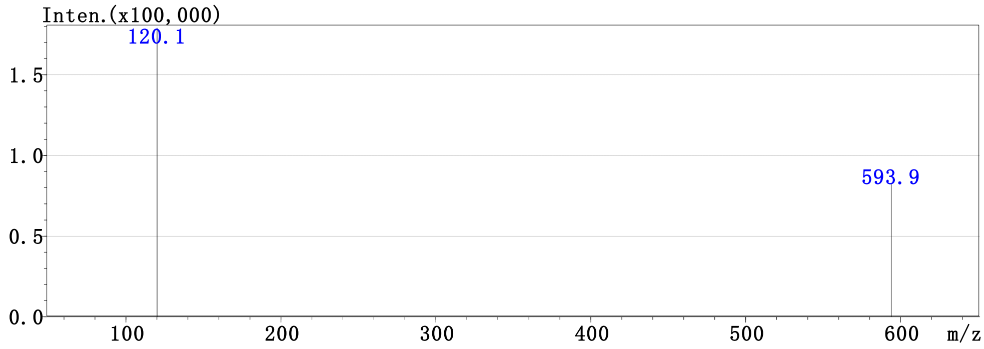

Figure S12. Product ion mass spectrum for confirming the product peak (**P1-P**) in Figure S10.

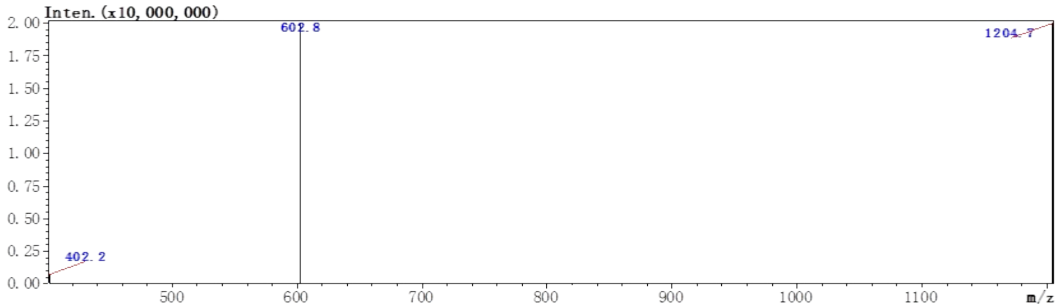

Figure S13. ESI MS spectrum of substrate peak (**P1**) in Figure S10.

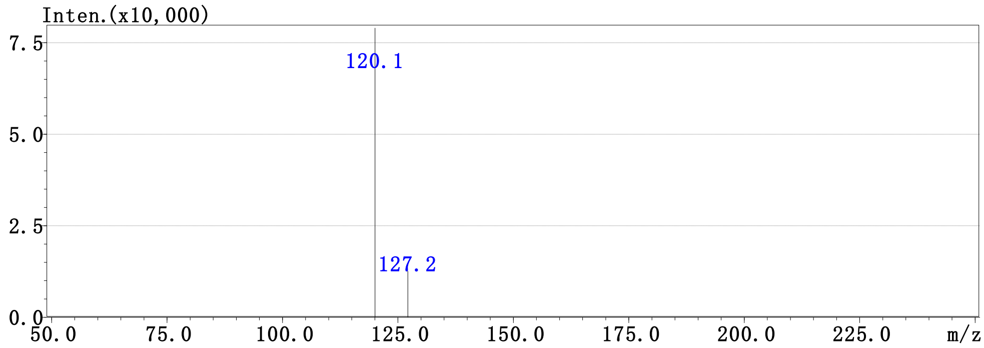

Figure S14. Product ion mass spectrum for confirming the substrate peak (**P1**) in Figure S10.

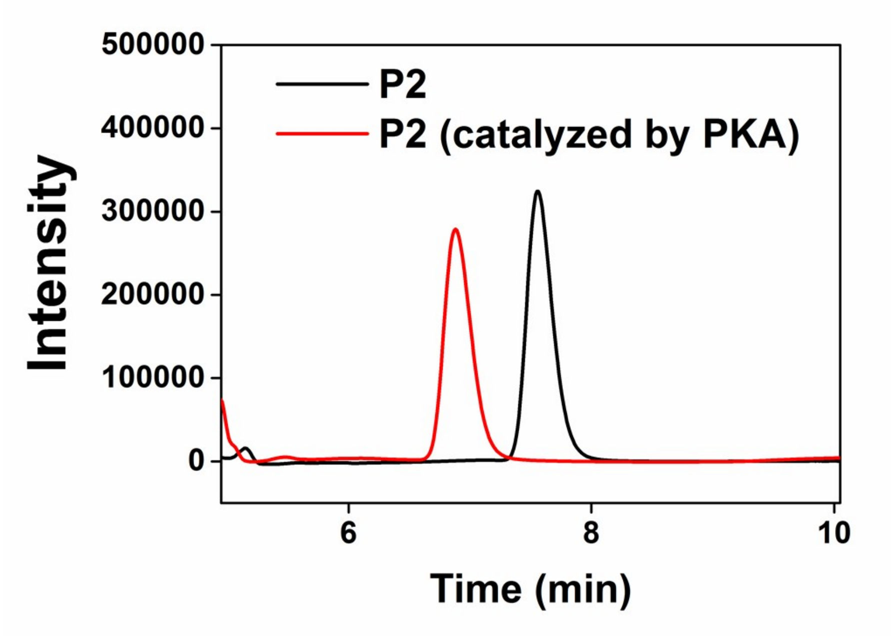

Figure S15. HPLC trace of **P2** before and after phosphorylated by PKA catalytic subunit for 4 days.

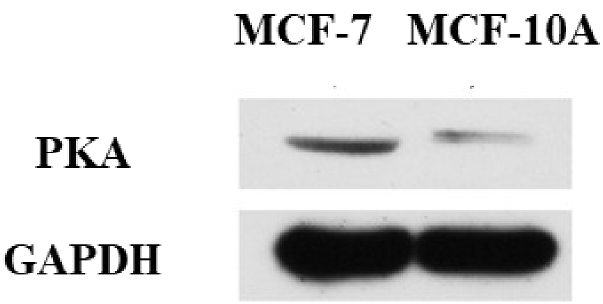

Figure S16. Expression level of PKA in MCF-7 Cells and MCF-10A cells determined by Western Blot.

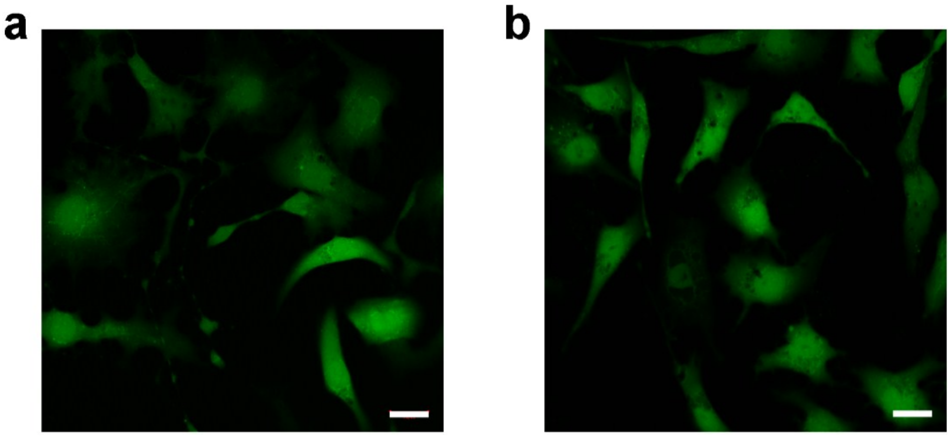

Figure S17. HUVEC cells were stained with cell tracker green (2,5-pyrrolidinedione) (a) without and (b) with **P2** (250 μg/mL) for 24 h. Scale bars: 20 μm.

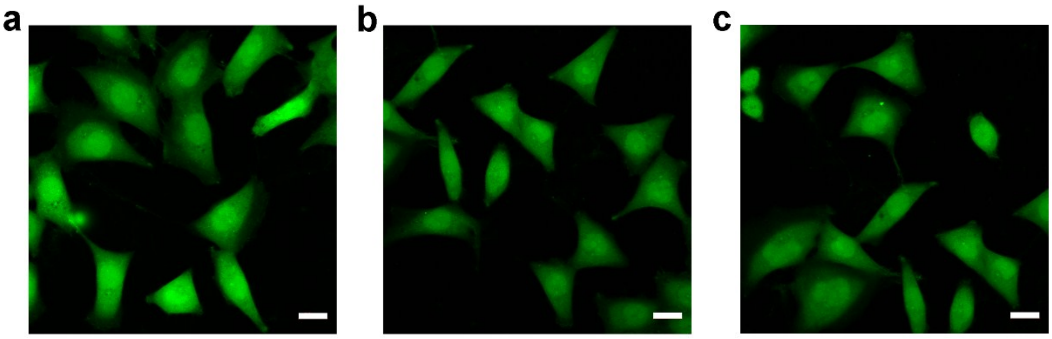

Figure S18. a, Normal MCF-7 cells stained with cell tracker green (2,5-pyrrolidinedione). b, MCF-7 cells were stained with cell tracker green (2,5-pyrrolidinedione) after treated with H89 (10 μg/mL) for 24 h. c, MCF-7 cells were stained with cell tracker green (2,5-pyrrolidinedione) after treated with H89 (10 μg/mL) for 24 h and then treated with **P2** (50 μg/mL) for 24 h. Scale bars: 20 μm.

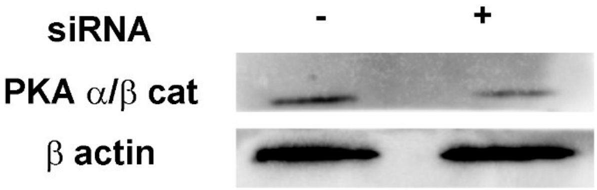

Figure S19. The expression level of PKA catalytic subunit in MCF-7 Cells without (left) or with (right) SignalSilence® PKA C-α siRNA I.

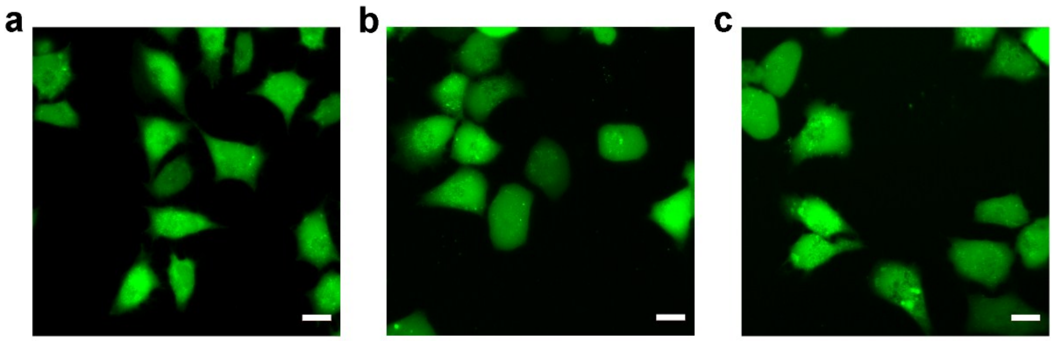

Figure S20. a, Normal MCF-7 cells stained with cell tracker green (2,5-pyrrolidinedione). b, PKA catalytic subunit downregulated MCF-7 cells stained with cell tracker green (2,5-pyrrolidinedione). c, PKA catalytic subunit downregulated MCF-7 cells stained with cell tracker green (2,5-pyrrolidinedione) after treated with **P2** (50 μg/mL) for 24 h. Scale bars: 20 μm.

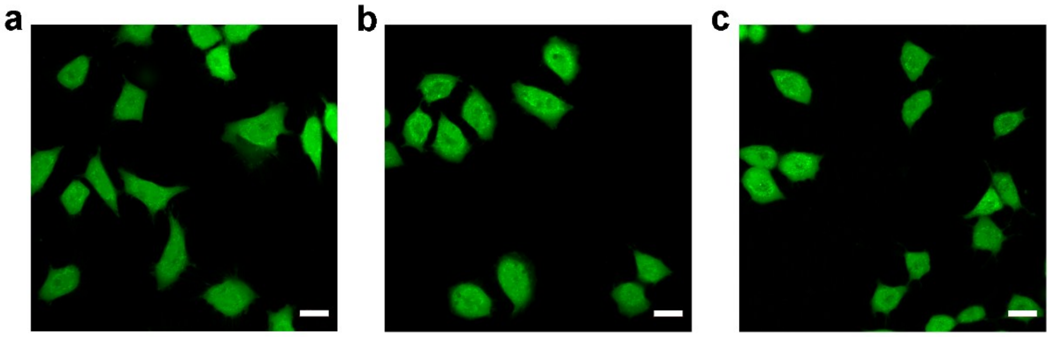

Figure S21. MCF-7 cells were stained with cell tracker green (2,5-pyrrolidinedione) (a) wild type; (b) incubated with **P1'** (50 μg/mL) for 24 h; (c) incubated with **P2'** (50 μg/mL) for 24 h. Scale bars: 20 μm.

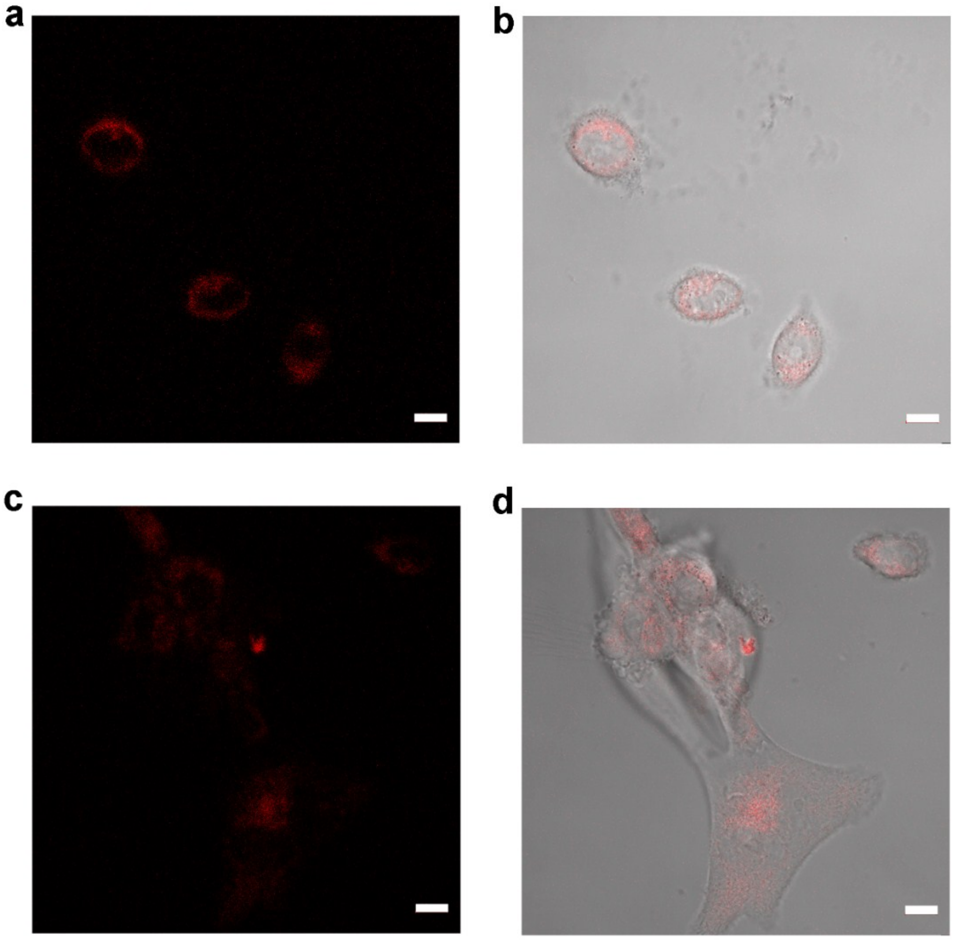

Figure S22. a, b Confocal images of MCF-7 cells treated with **P2-Cy7** (200 μg/mL). c, d Confocal images of MCF-10A cells treated with **P2-Cy7** (200 μg/mL). Scale bars: 10 μm.

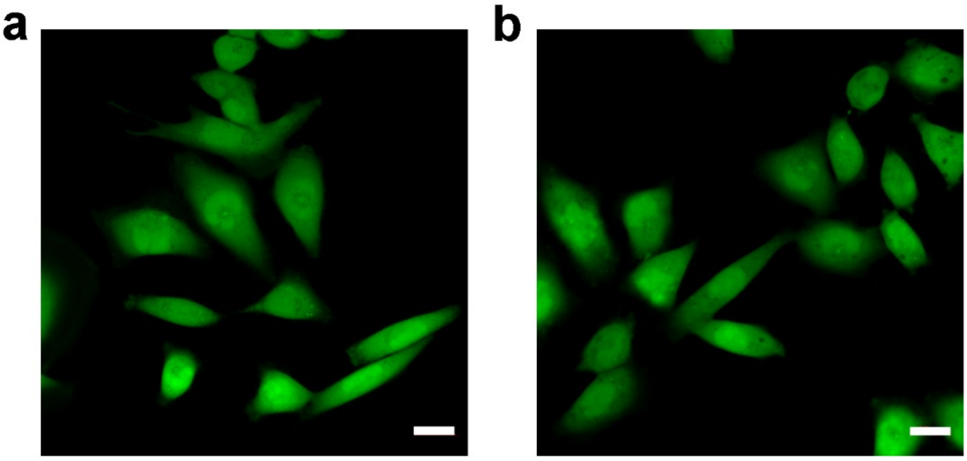

Figure S23. MCF-7 cells were stained with cell tracker green (2,5-pyrrolidinedione) (a) without and (b) with **P3** (50 μg/mL) for 24 h. Scale bars: 20 μm.

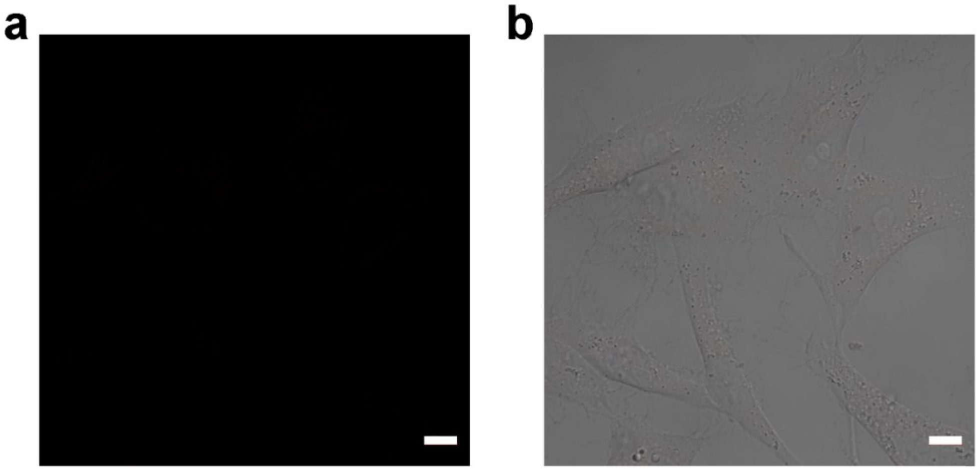

Figure S24. Confocal images of MCF-7 cells treated with **P3-Cy7** (50 μg/mL) for 24 h. Scale bars: 10 μm.

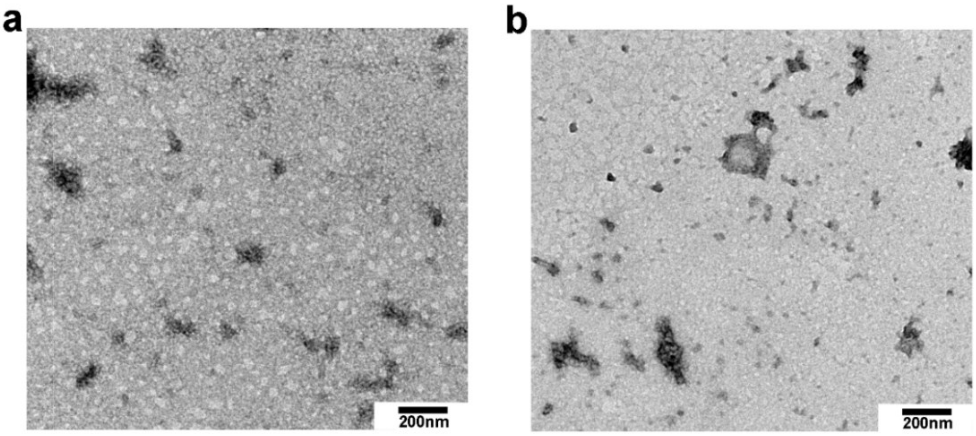

Figure S25. (a) TEM image of fraction of MCF-7 cells pre-treated with 50 μg/mL **P1**. (b) TEM image of fraction of MCF-7 cells pre-treated with 50 μg/mL **P1-P**.

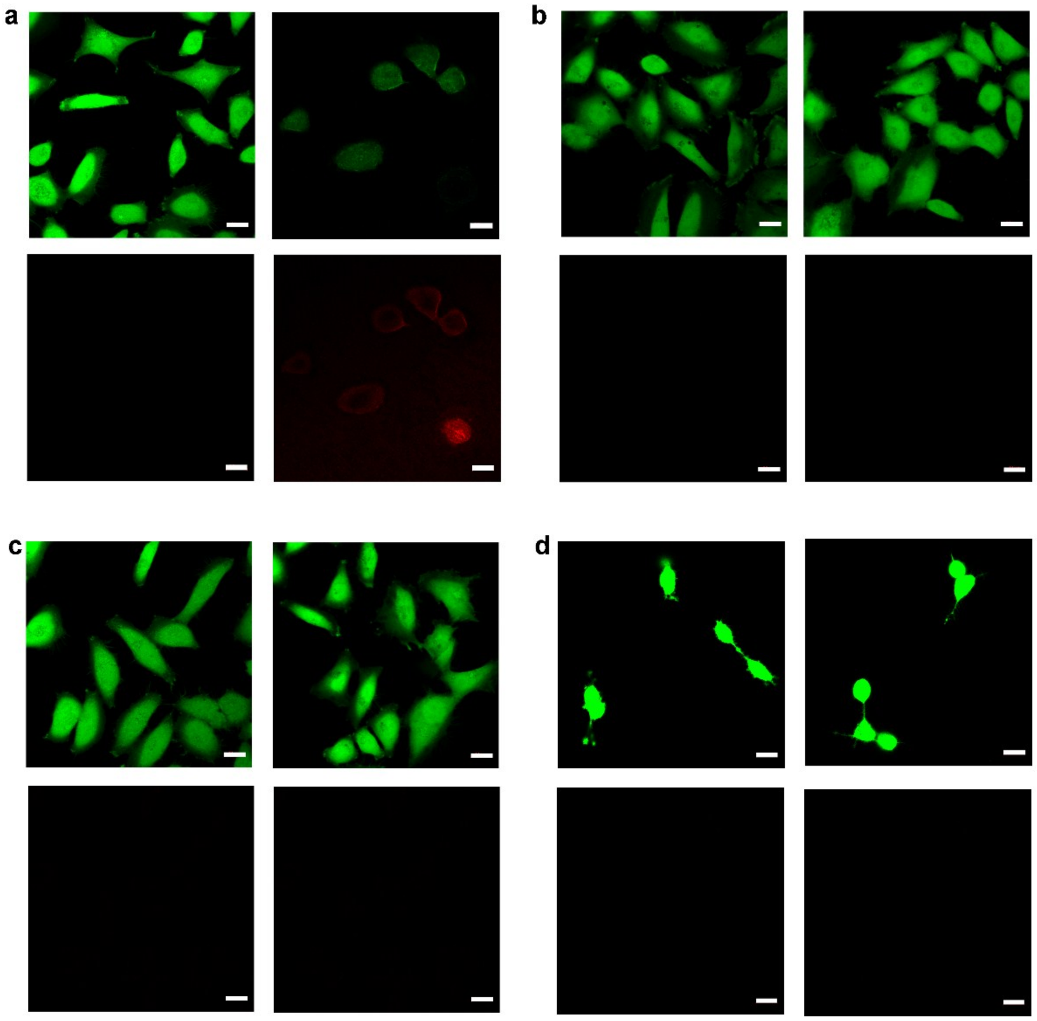

Figure S26. Confocal images of LIVE/DEAD Cell Imaging Kit stained (a) MCF-7 cells incubated (left) without or (right) with **P2** (100 μg/mL) for 24 h. (b) MCF-7 cells incubated (left) without or (right) with **P1** (100 μg/mL) for 24 h. (c) MCF-7 cells incubated (left) without or (right) with **P3** (100 μg/mL) for 24 h. (d) MCF-10A cells incubated (left) without or (right) with **P2** (100 μg/mL) for 24 h. Scale bar: 20 μm.

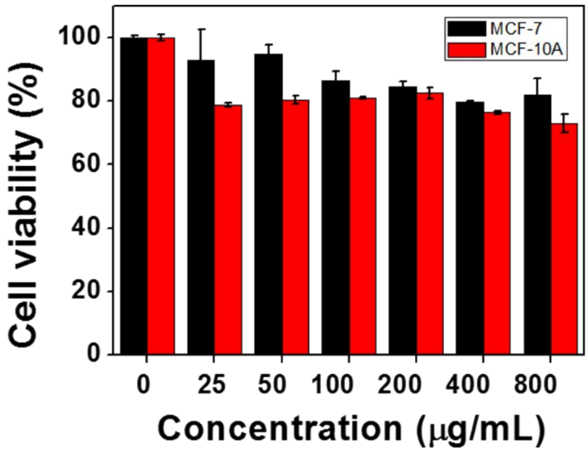

Figure S27. Cell viability of MCF-7 and MCF-10A cells cultured with **P2** for 72 h.

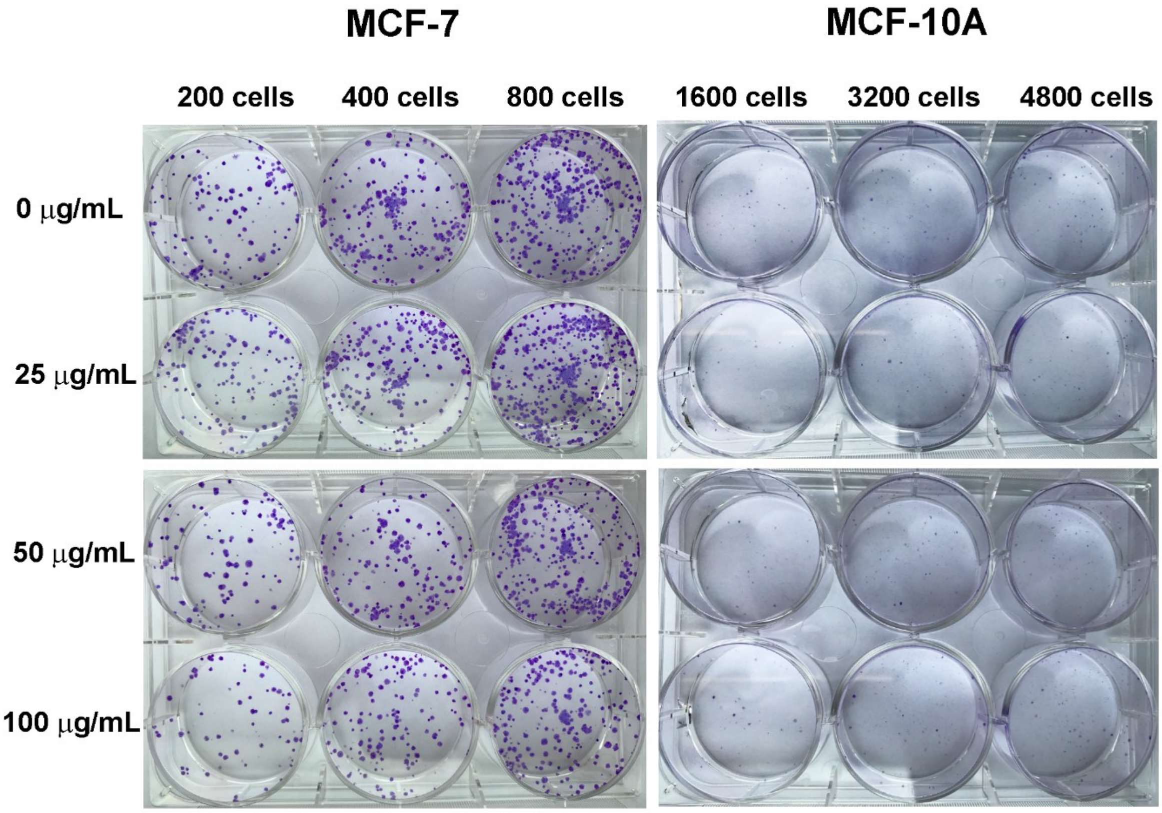

Figure S28. Cell clones produced by **P2** (0, 25, 50, 100 μg/mL) treated MCF-7 and MCF-10A cells.

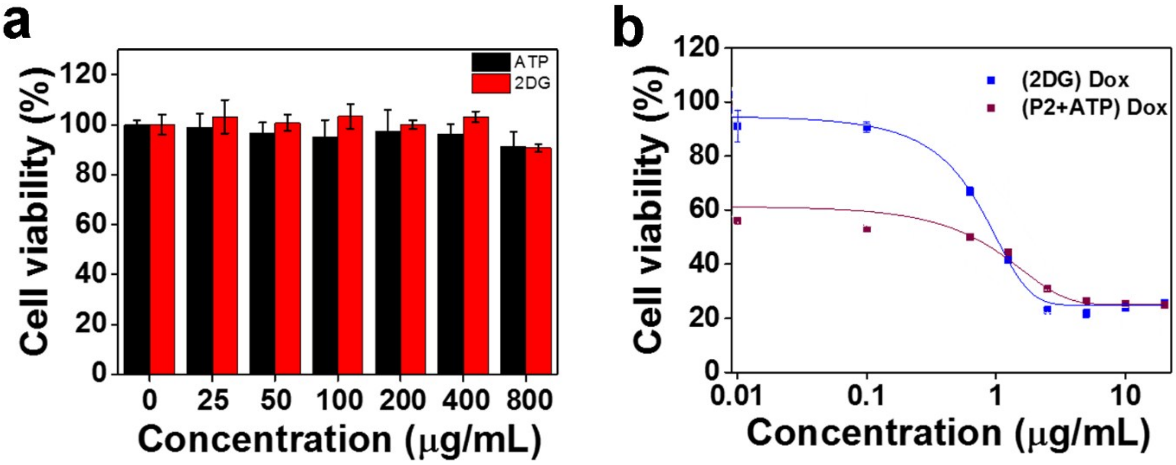

Figure S29. a, Cell viability of MCF-7 cells cultured with ATP or 2DG for 24 h. b, Cell viability of MCF-7 cells against (**P2**+ATP) Dox, (2DG) Dox. (ATP: 200 μg/mL, 2DG: 100 μg/mL).

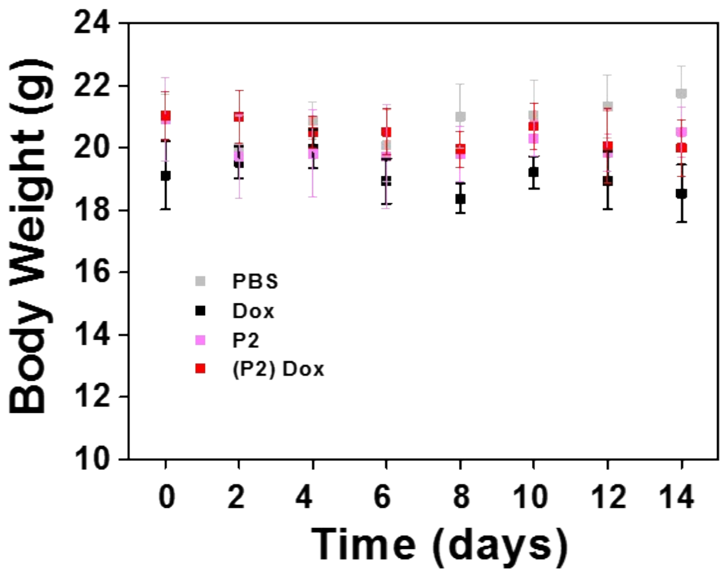

Figure S30. Body weight change curves of MCF-7 tumor-bearing mice treated with PBS, **P2**, Dox, (**P2)** Dox, respectively.

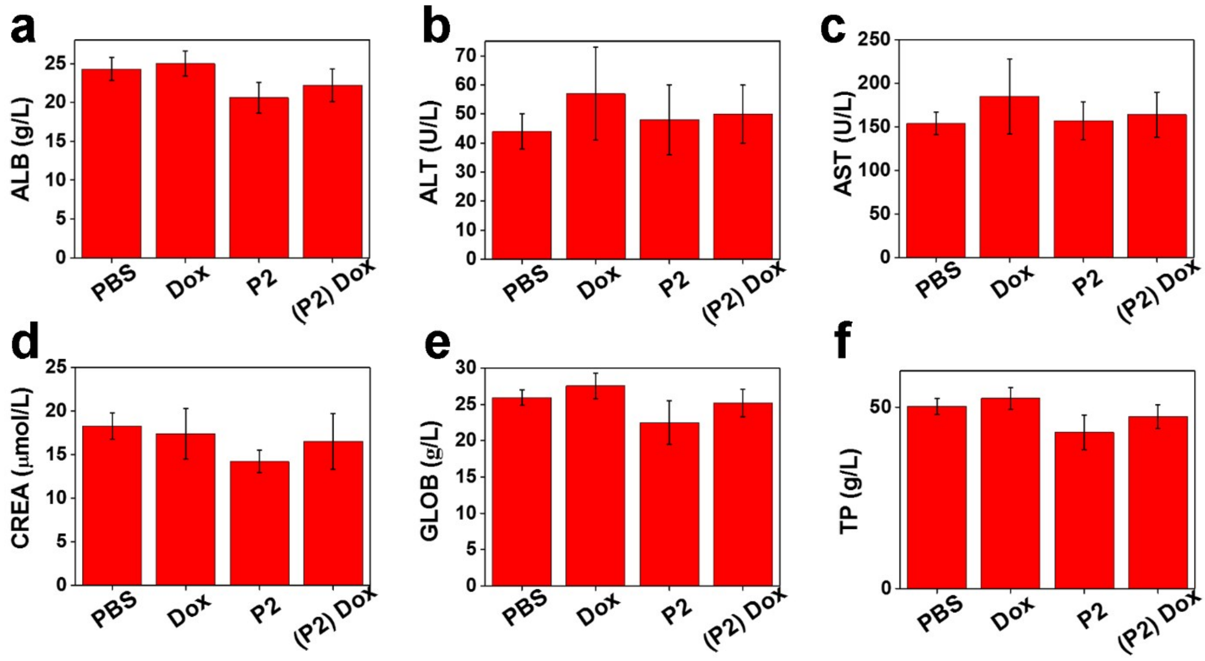

Figure S31. Serum biochemical indexes including ALB (a), ALT (b), AST (c), CREA (d), GLOB (e), TP (f) of the MCF-7 tumor-bearing mice treated with PBS, **P2**, Dox, (**P2)** Dox, respectively.

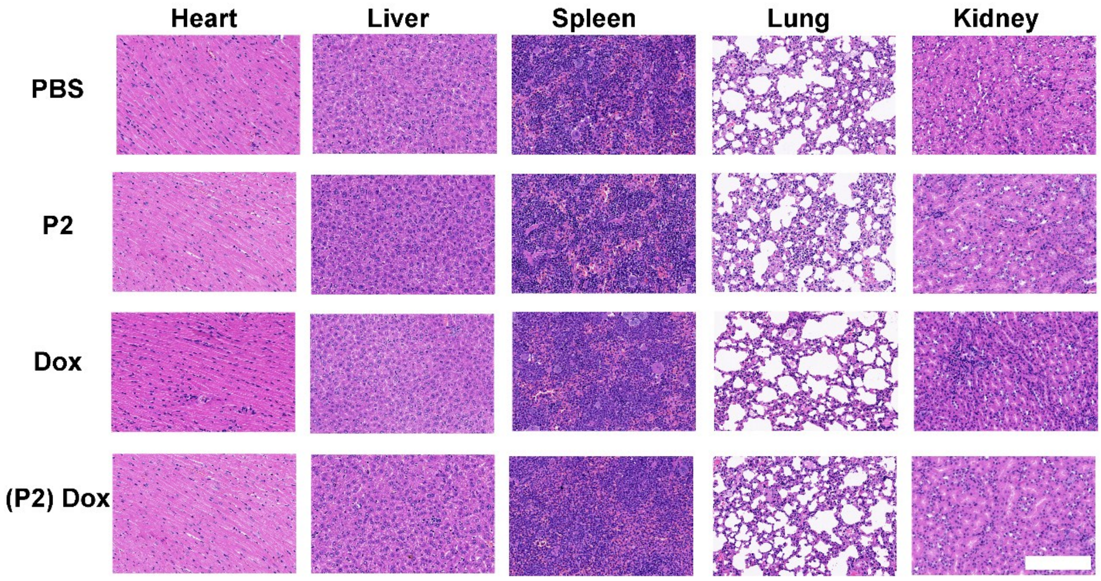

Figure S32. HE staining of the main organs including heart, liver, spleen, lung, and kidney after treated with PBS, **P2**, Dox, (**P2)** Dox, respectively. Scale bar: 200 μm.

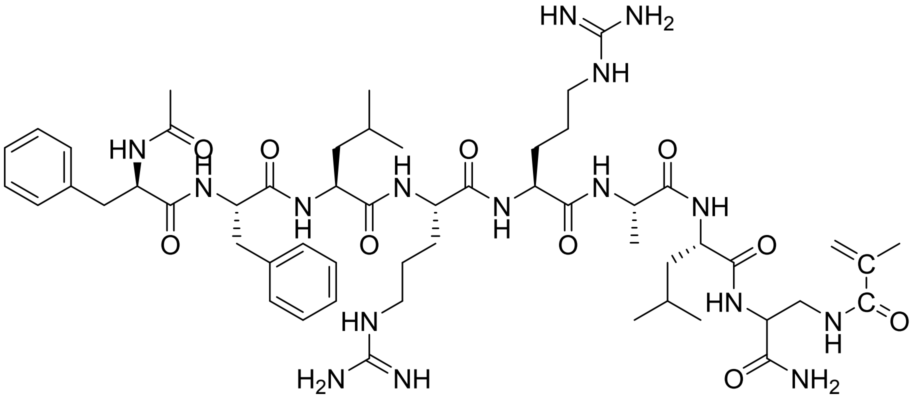

Figure S33. Molecular structure of **P1'** (without serine).

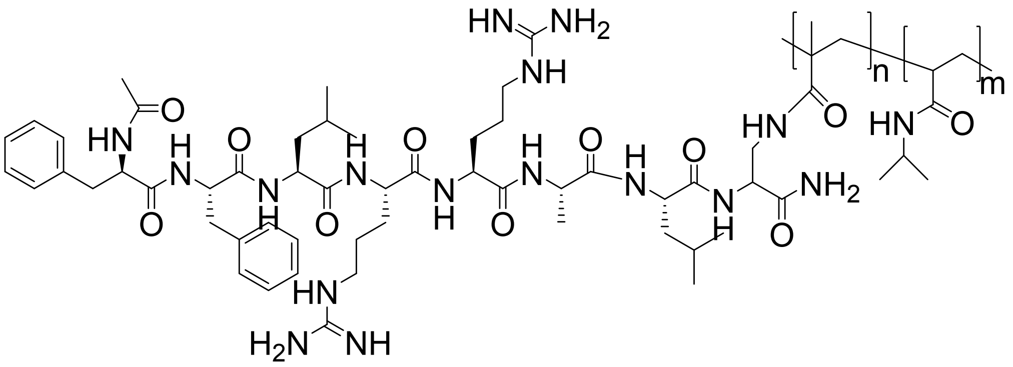

Figure S34. Molecular structure of **P2'** (without serine).

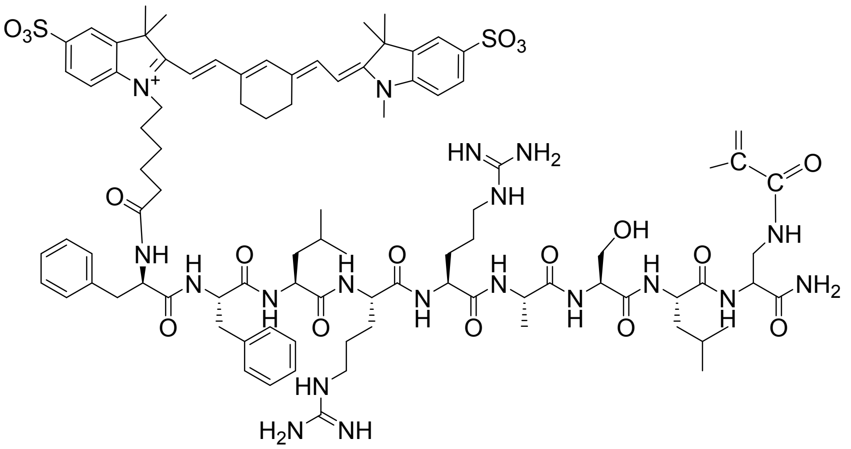

Figure S35. Molecular structure of **P1**-Cy7.

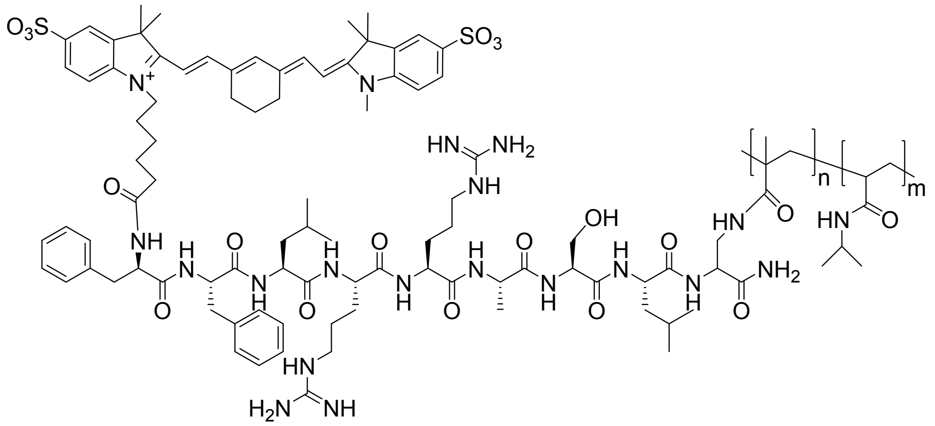

Figure S36. Molecular structure of **P2**-Cy7.

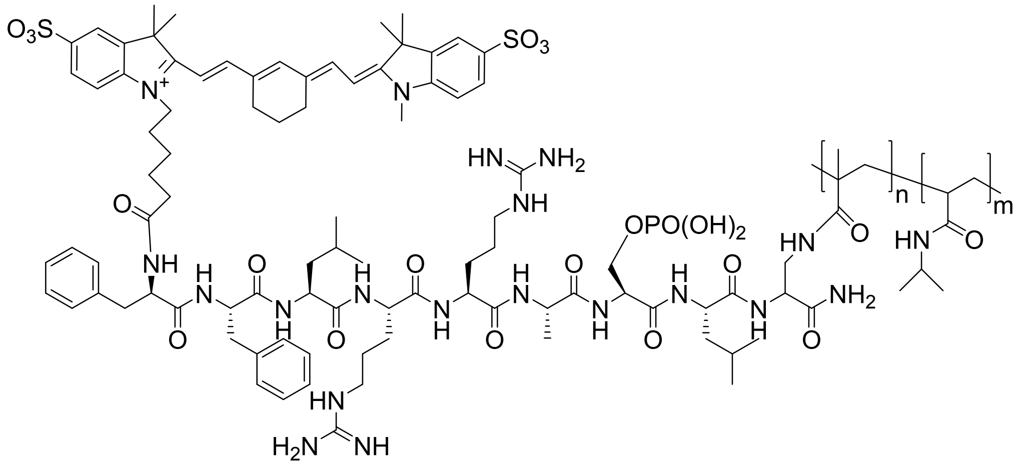

Figure S37. Molecular structure of **P3**-Cy7.

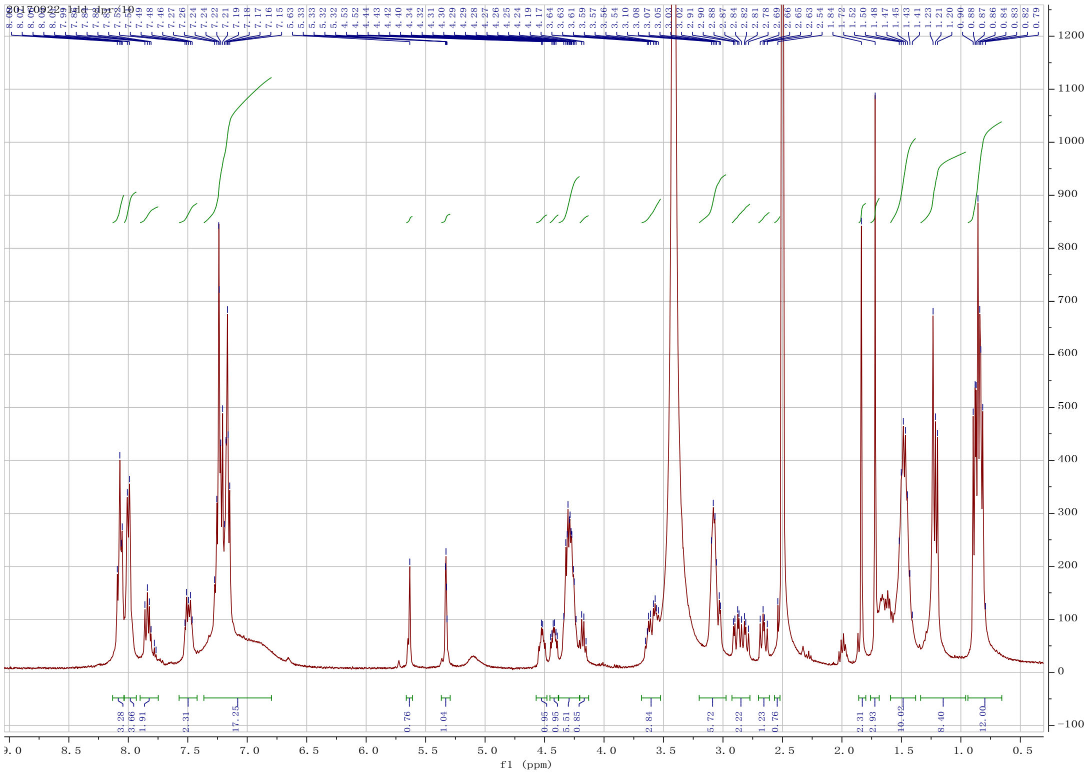

Figure S38. ^{1}H-NMR spectrum of **P1**.

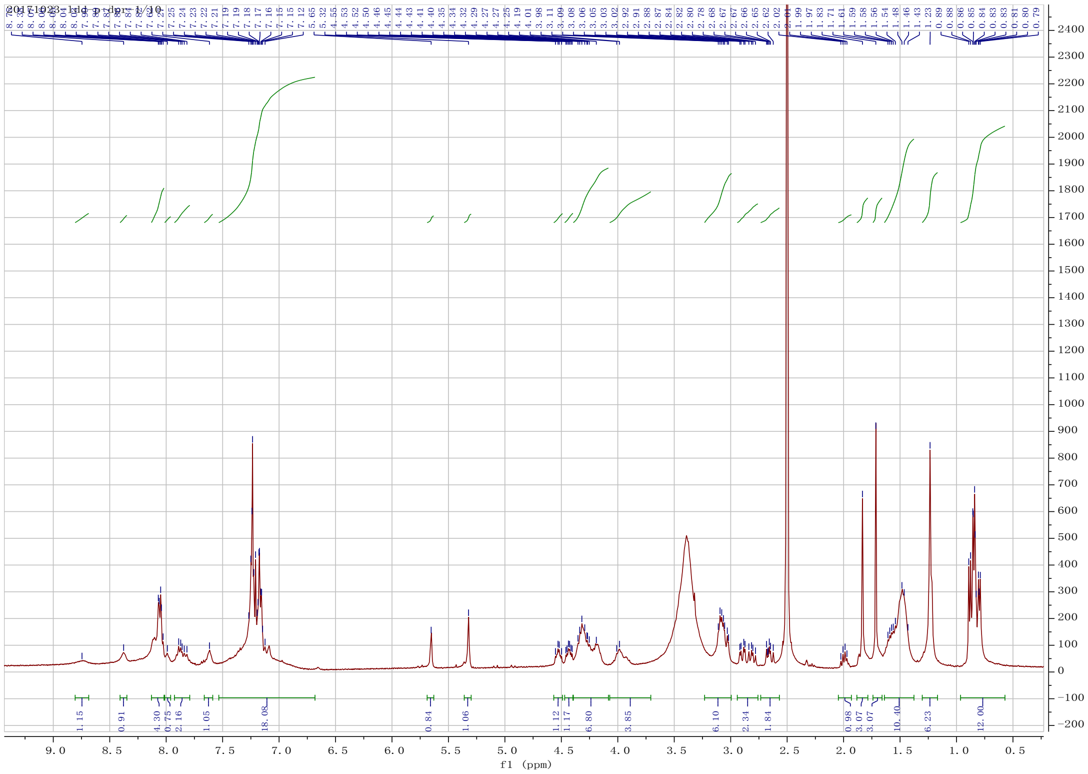

Figure S39. ^{1}H-NMR spectrum of **P1-P**.

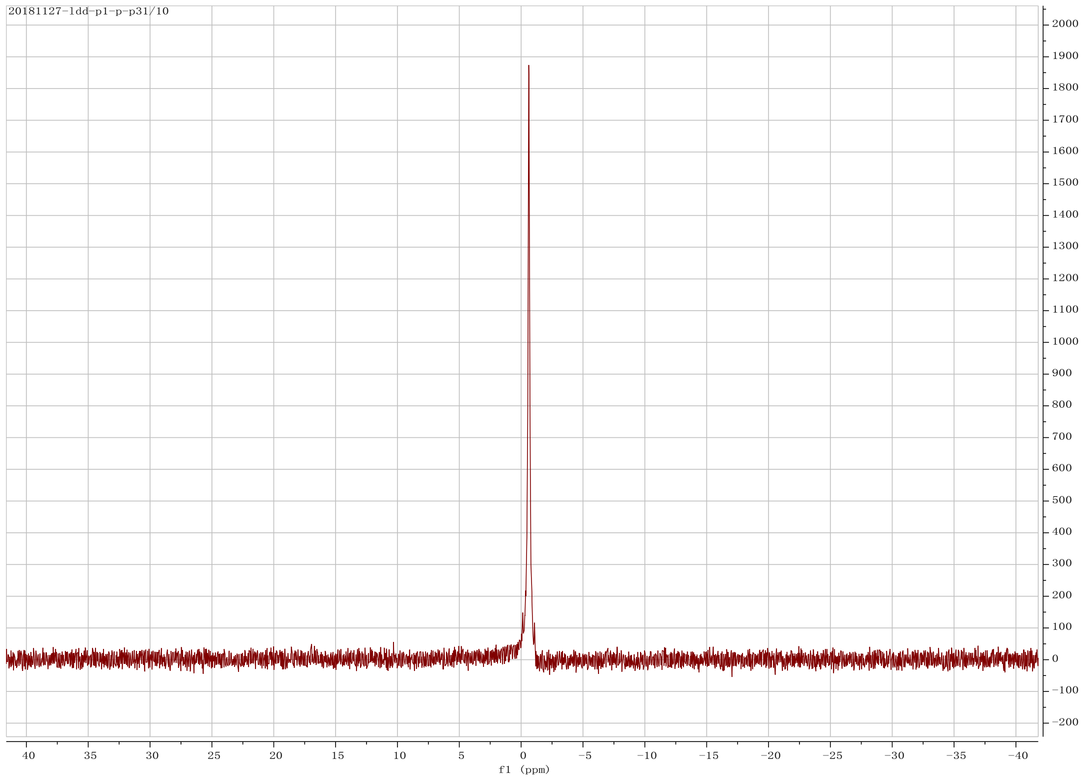

Figure S40. ^{31}P-NMR spectrum of **P1-P**.

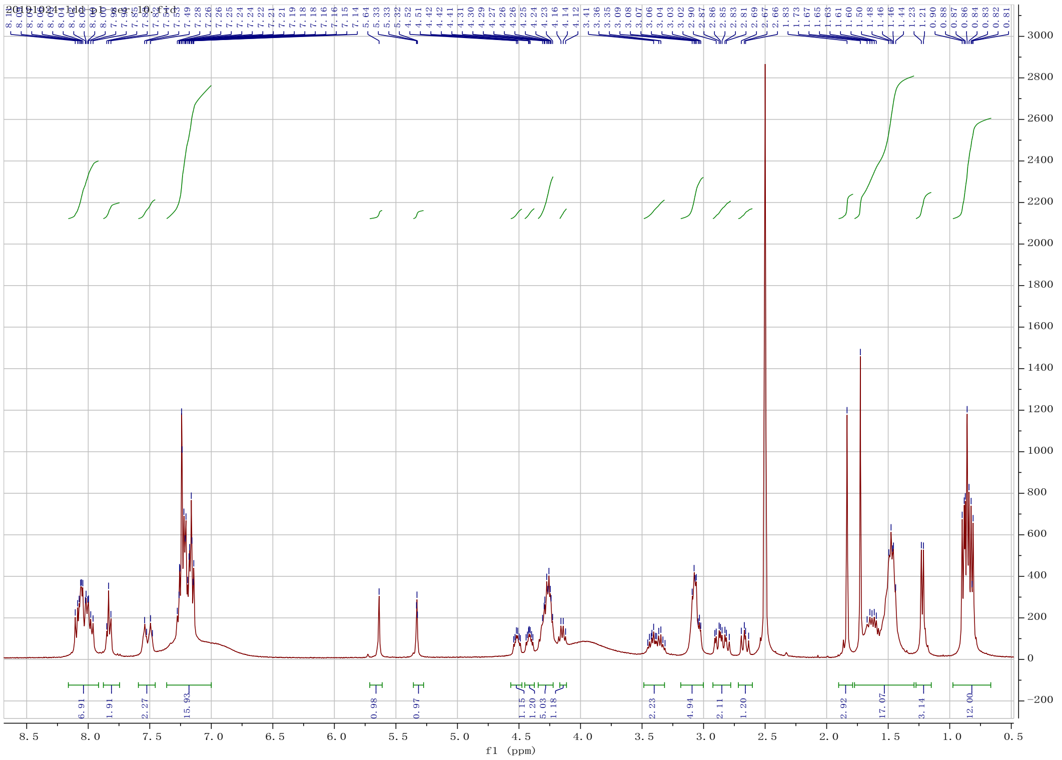

Figure S41. ^{1}H-NMR spectrum of **P1’** (without serine).

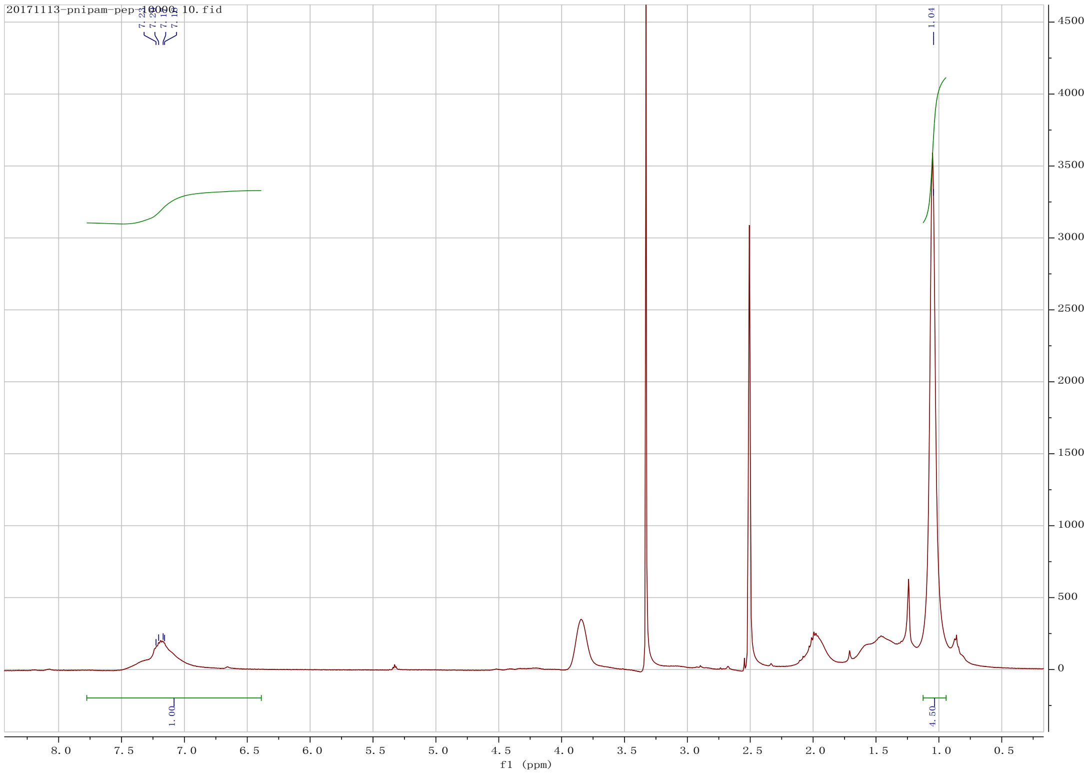

Figure S42. ^{1}H-NMR spectrum of **P2**.

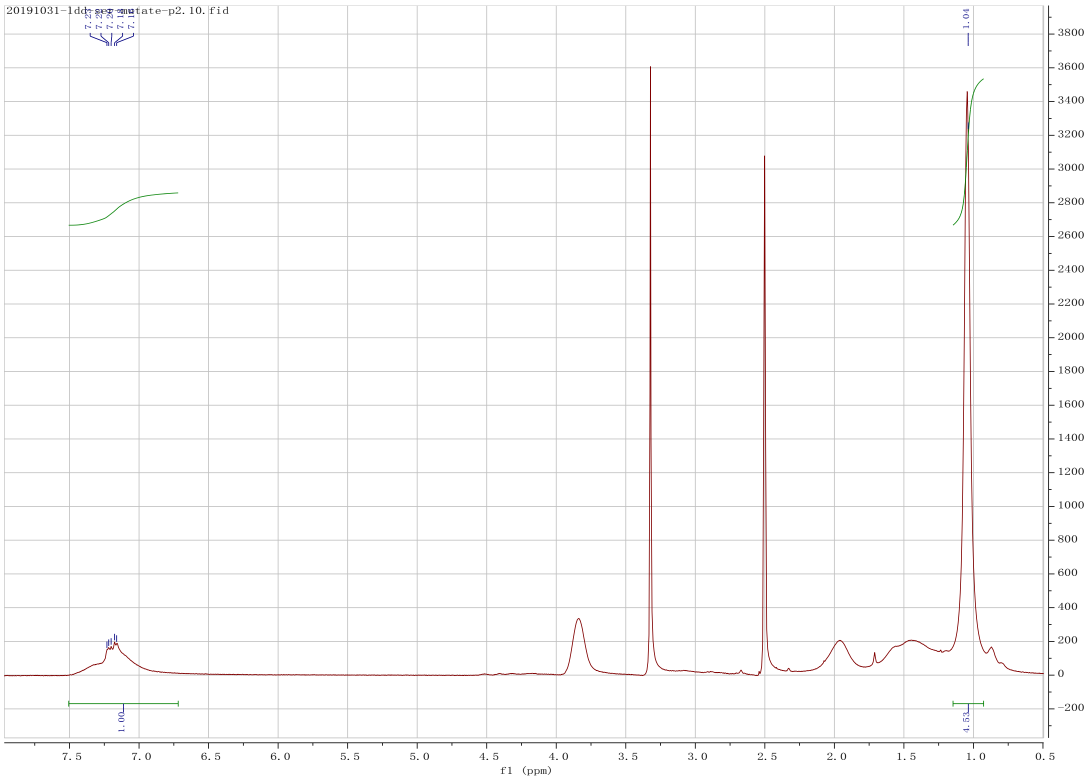

Figure S43. ^{1}H-NMR spectrum of **P2’** (without serine).

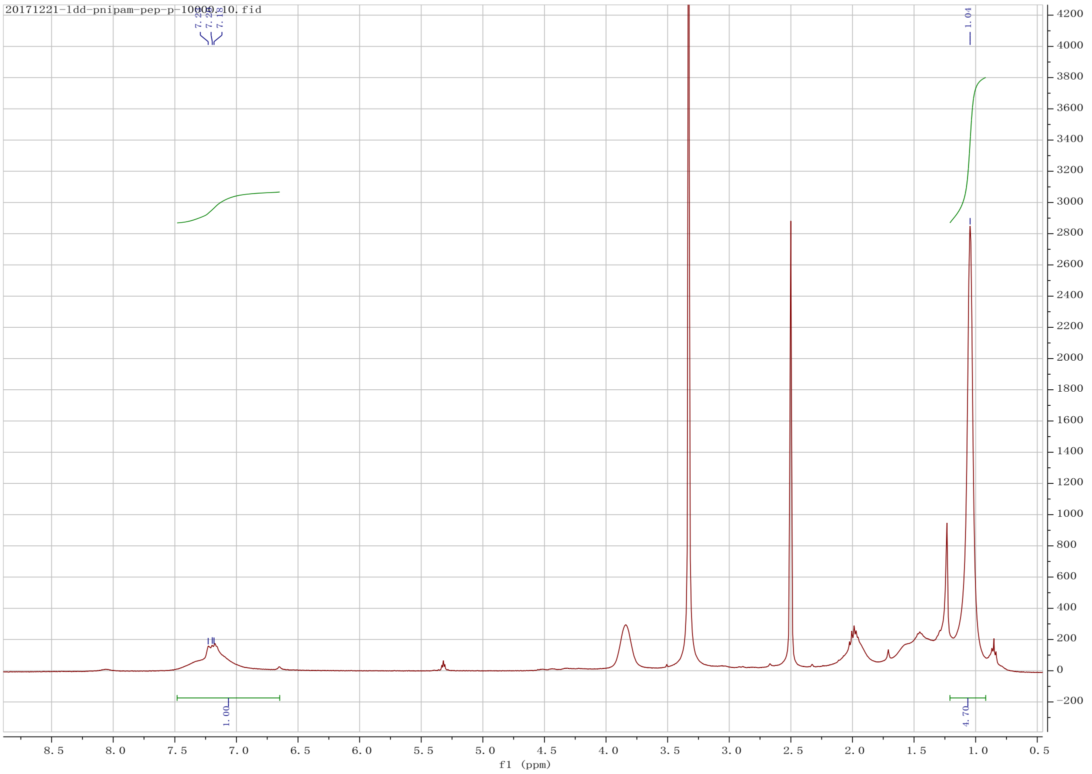

Figure S44. ^{1}H-NMR spectrum of **P3**.

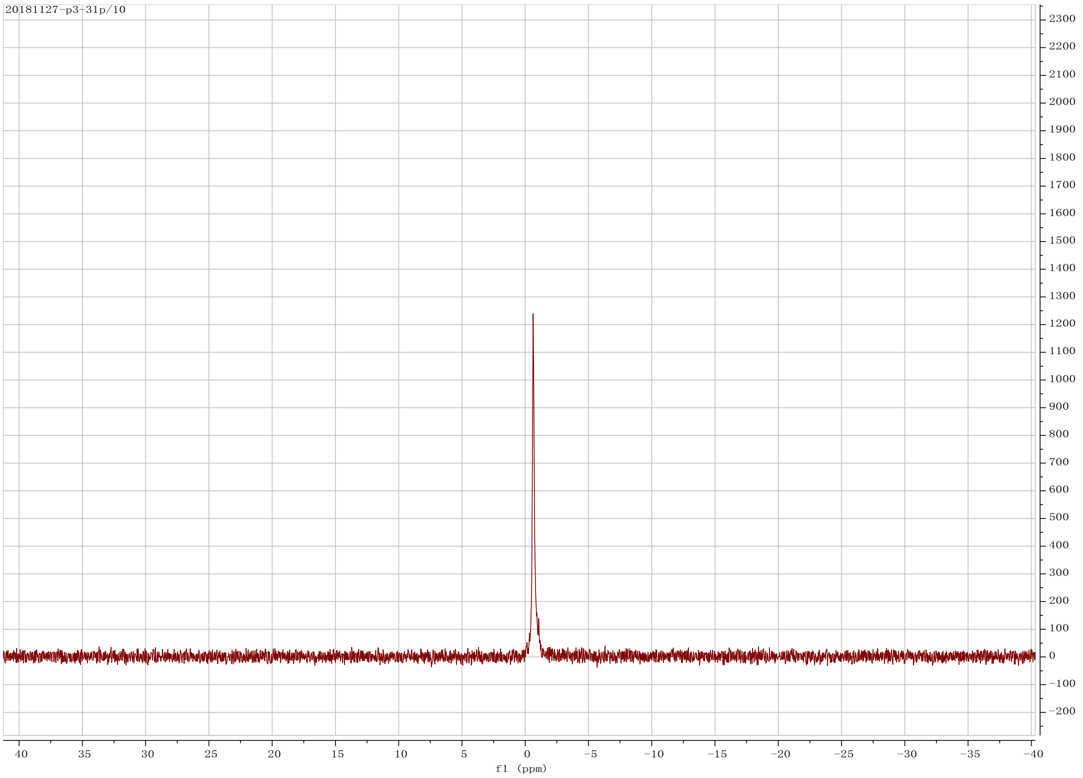

Figure S45. ^{31}P-NMR spectrum of **P3**.

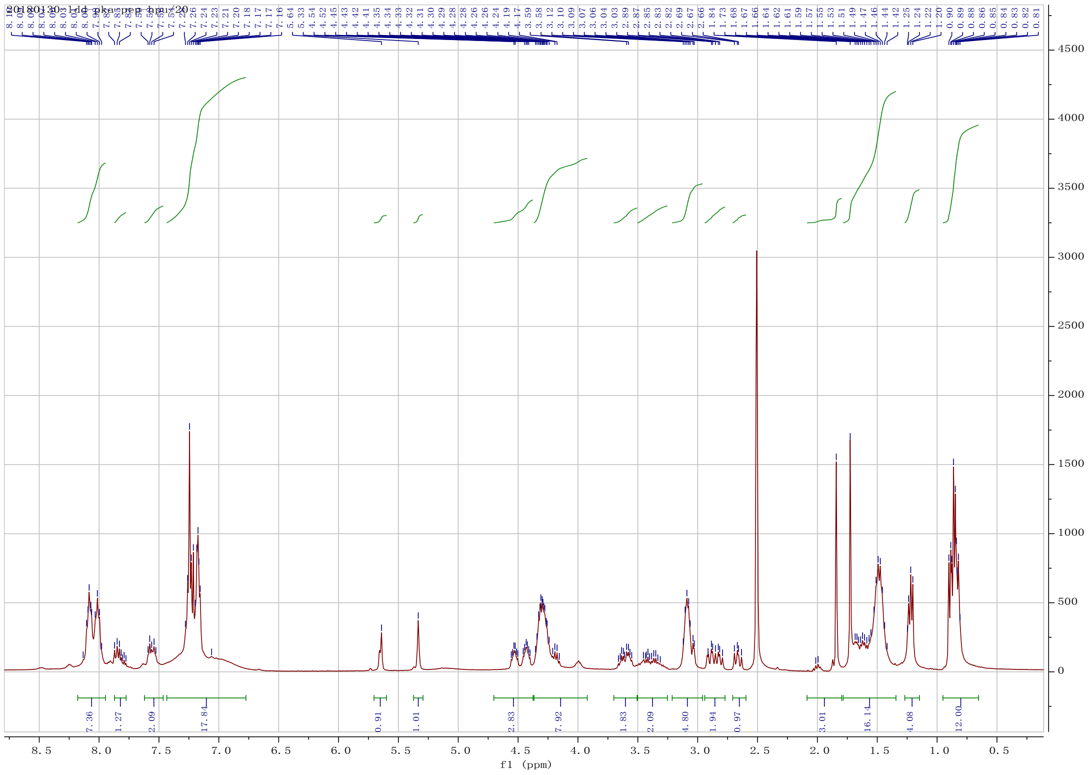

Figure S46. ^{1}H-NMR spectrum of the **P1** phosphorylated by PKA.

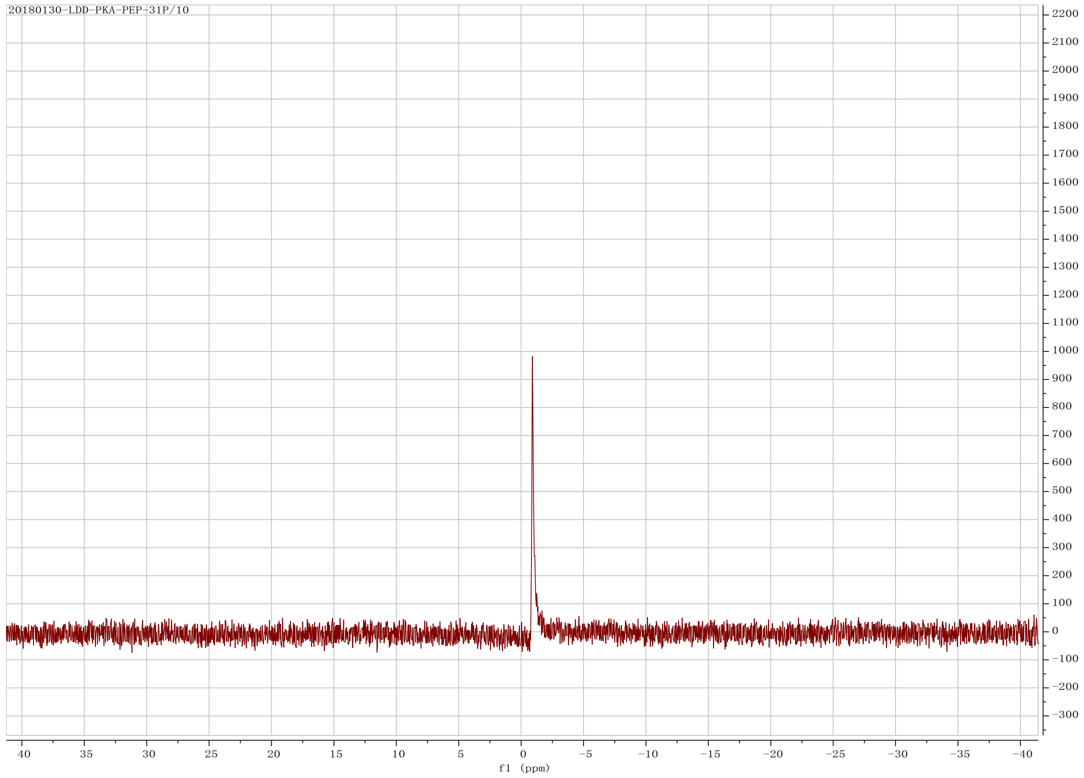

Figure S47. ^{31}P-NMR spectrum of the **P1** phosphorylated by PKA.

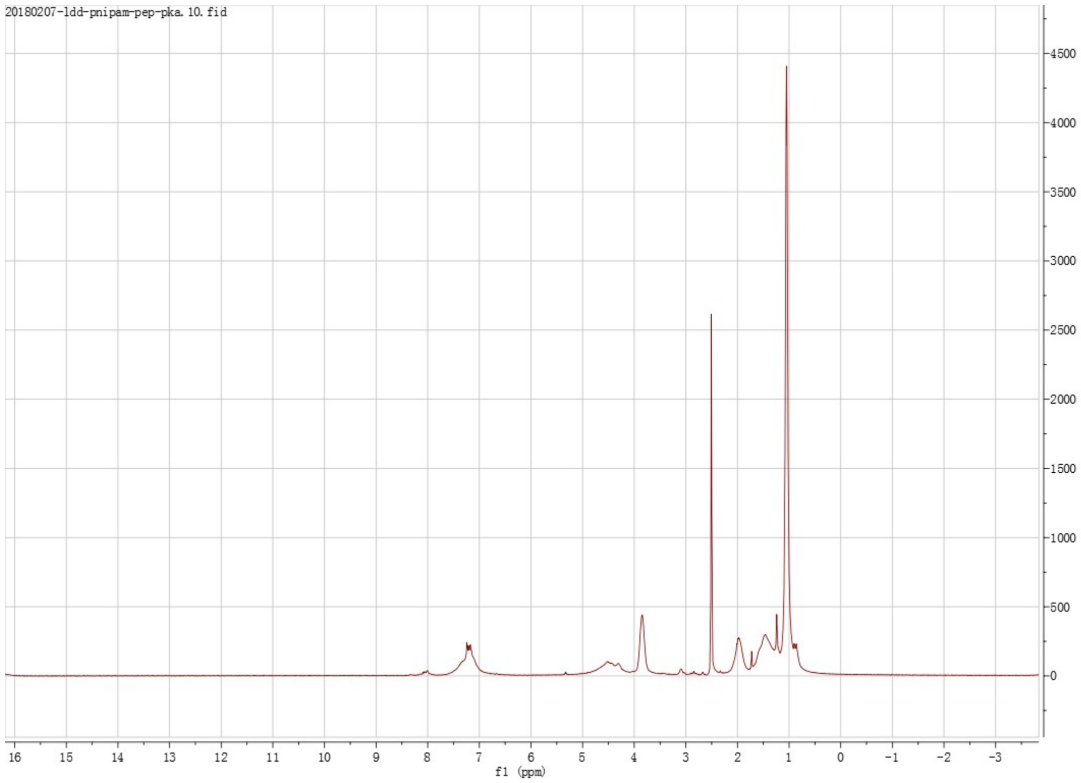

Figure S48. ¹H-NMR spectrum of the **P2** phosphorylated by PKA.

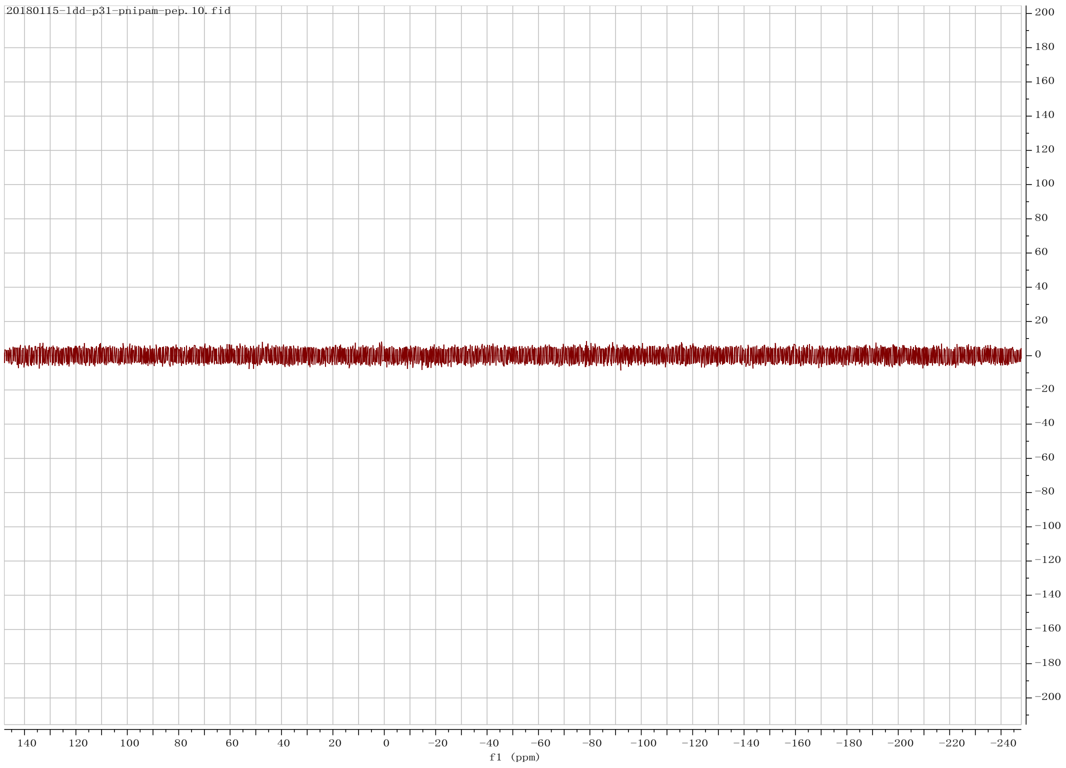

Figure S49. ³¹P-NMR spectrum of the **P2** before phosphorylated by PKA.

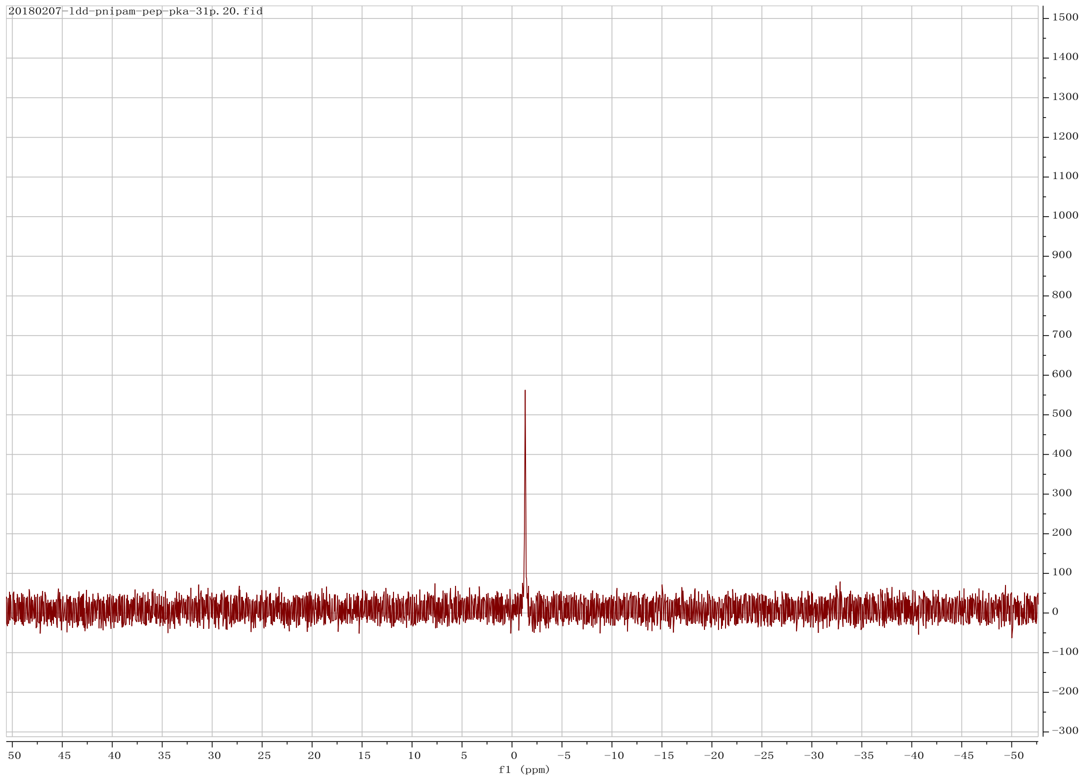

Figure S50. ^{31}P-NMR spectrum of the **P2** phosphorylated by PKA and purified by HPLC.

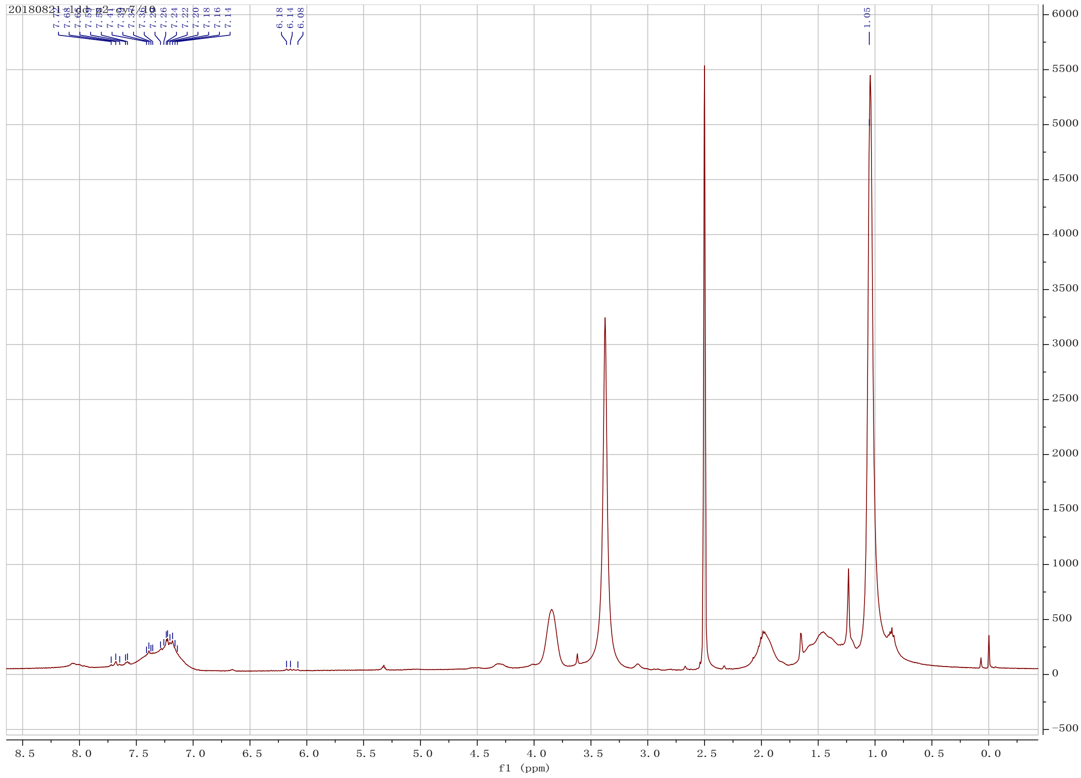

Figure S51. ^{1}H-NMR spectrum of **P2**-Cy7.

Table S1. The concentration of **P1-P** and **P1** found in MCF-7 and MCF-10A cells with the addition of **P1** (1 mg/mL) for 24 h.

	**Peptides**	
**Cell lines**	**P1** (ppb)	**P1-P** (ppb)
**MCF-7**	238.89 ± 3.00	11.59 ± 0.36
**MCF-10A**	124.48 ± 2.12	2.59 ± 0.13

Table S2. Relative quantification of **P2-Cy7** (50 μg/mL, 24 h) and **P3-Cy7** (50 μg/mL, 24 h) in MCF-7 and MCF-10A cells by fluorescence intensity.

	**Fluorescence intensity mean value (count/nm^2)**	
**Cell lines**	**P2-Cy7**	**P3-Cy7**
**MCF-7**	1617.44 ± 41.83	231.85 ± 24.95
**MCF-10A**	1031.89 ± 43.74	106.05 ± 9.07

Table S3. Clonogenic assay of MCF-7 cells treated with **P2** (25, 50, 100 μg/mL) for 24 h and MCF-10A cells treated with **P2** (25, 50, 100 μg/mL) for 24 h.

**Cell lines**	**PE**	**P2 (μg/mL)**	**SF**
**MCF-7**	80% ± 0.04	25	76% ± 0.03
		50	66% ± 0.04
		100	43% ± 0.01
**MCF-10A**	9% ± 0.01	25	96% ± 0.01
		50	94% ± 0.02
		100	92% ± 0.05
